# Open and reusable annotated mass spectrometry dataset of a chemodiverse collection of 1,600 plant extracts

**DOI:** 10.1093/gigascience/giac124

**Published:** 2023-01-18

**Authors:** Pierre-Marie Allard, Arnaud Gaudry, Luis-Manuel Quirós-Guerrero, Adriano Rutz, Miwa Dounoue-Kubo, Tom W N Walker, Emmanuel Defossez, Christophe Long, Antonio Grondin, Bruno David, Jean-Luc Wolfender

**Affiliations:** Institute of Pharmaceutical Sciences of Western Switzerland, University of Geneva, 1211 Geneva, Switzerland; School of Pharmaceutical Sciences, University of Geneva, 1211 Geneva, Switzerland; Department of Biology, University of Fribourg, 1700 Fribourg, Switzerland; Institute of Pharmaceutical Sciences of Western Switzerland, University of Geneva, 1211 Geneva, Switzerland; School of Pharmaceutical Sciences, University of Geneva, 1211 Geneva, Switzerland; Institute of Pharmaceutical Sciences of Western Switzerland, University of Geneva, 1211 Geneva, Switzerland; School of Pharmaceutical Sciences, University of Geneva, 1211 Geneva, Switzerland; Institute of Pharmaceutical Sciences of Western Switzerland, University of Geneva, 1211 Geneva, Switzerland; School of Pharmaceutical Sciences, University of Geneva, 1211 Geneva, Switzerland; Faculty of Pharmaceutical Sciences, Tokushima Bunri University, 770-8514 Tokushima, Japan; Institute of Biology, University of Neuchâtel, 2000 Neuchâtel, Switzerland; Department of Biology, University of Fribourg, 1700 Fribourg, Switzerland; Institute of Biology, University of Neuchâtel, 2000 Neuchâtel, Switzerland; Direction Scientifique Naturactive, Pierre Fabre Medicament, 81100 Castres, France; Green Mission Pierre Fabre, Institut de Recherche Pierre Fabre, 31562 Toulouse, France; Green Mission Pierre Fabre, Institut de Recherche Pierre Fabre, 31562 Toulouse, France; Institute of Pharmaceutical Sciences of Western Switzerland, University of Geneva, 1211 Geneva, Switzerland; School of Pharmaceutical Sciences, University of Geneva, 1211 Geneva, Switzerland

**Keywords:** plant extracts collection, metabolomics, drug discovery, LC-MS, natural products, mass spectrometry, chemodiversity, open science, biodiversity digitization

## Abstract

As privileged structures, natural products often display potent biological activities. However, the discovery of novel bioactive scaffolds is often hampered by the chemical complexity of the biological matrices they are found in. Large natural extract collections are thus extremely valuable for their chemical novelty potential but also complicated to exploit in the frame of drug-discovery projects. In the end, it is the pure chemical substances that are desired for structural determination purposes and bioactivity evaluation. Researchers interested in the exploration of large and chemodiverse extract collections should thus establish strategies aiming to efficiently tackle such chemical complexity and access these structures. Establishing carefully crafted digital layers documenting the spectral and chemical complexity as well as bioactivity results of natural extracts collections can help prioritize time-consuming but mandatory isolation efforts. In this note, we report the results of our initial exploration of a collection of 1,600 plant extracts in the frame of a drug-discovery effort. After describing the taxonomic coverage of this collection, we present the results of its liquid chromatography high-resolution mass spectrometric profiling and the exploitation of these profiles using computational solutions. The resulting annotated mass spectral dataset and associated chemical and taxonomic metadata are made available to the community, and data reuse cases are proposed. We are currently continuing our exploration of this plant extract collection for drug-discovery purposes (notably looking for novel antitrypanosomatids, anti-infective and prometabolic compounds) and ecometabolomics insights. We believe that such a dataset can be exploited and reused by researchers interested in computational natural products exploration.

## Data Description

In the frame of a partnership between academia and industry, a collaboration was established in 2017 between the Laboratory of Phytochemistry and Bioactive Natural Products at the University of Geneva (LPBNP), Switzerland, and the Pierre Fabre Laboratories (PFL) in Toulouse, France. This collaboration had the objective to explore and exploit the chemodiversity of a large collection of plant extracts (furnished by the industrial partner) using state-of-the-art mass spectrometric profiling methods and associated data-mining solutions (performed and developed by the academic partner). The main goal of this research project is to establish a precise and exploitable description of the wide chemical diversity displayed in the plant extracts collection in order to orient further isolation efforts aiming to discover, in a first move, novel antitrypanosomatid scaffolds and deepen chemotaxonomic knowledge of such a large set.

### Context

Pierre Fabre Laboratories were founded in 1962 by Pierre Fabre, a French pharmacist from Castres (Tarn, France). They have specialized since the very beginning in the exploration and valorization of plants as medicines, health, and beauty products. Innovation based on the vegetal world in PFL encompasses chemotherapy treatments with vinca alkaloids extracted from the leaves of *Catharanthus roseus* (Q161093) [1], dermo-cosmetic products such as hair dyes from *Lawsonia inermis* (Q182448) extracts [2], or celastrol (Q5057534)–enriched extracts for psoriatic skin obtained from *in vitro* plant cell culture of *Tripterygium wilfordii* (Q1424919) [3]. In 1998, Pierre Fabre decided to launch a high-throughput screening (HTS) program based on plant extracts with the objective to find novel anticancer drugs. The first samples of plant parts were collected in 1998, and when the HTS program ended in December 2015, the collection contained just over 17,000 unique samples. Multiple scientific articles originating from the exploration of this unique collection have been published over those years [4–7]. Hereafter is a selection of natural products–based scaffolds that entered antitumor medicinal chemistry programs: dimeric derivatives of artemisinin (Q426921) [8], flavagline derivatives (Q3073444) [9], griseofulvin (Q416096) [10], narciclasine (Q18379239) and pancratistatin (Q7130395) [11], neoboutomellerone [12], and triptolide (Q906351) [13].

The PFL collection, which is among the largest collection of plant samples worldwide with over 17,000 unique samples, was registered on 1 April 2020 at the European Commission under the accession number 03-FR-2020. This official registration recognizes the legality of the access and management process. It means that the collection meets the criteria set out in the European Access and Benefit Sharing Regulation, which implements at the European level the requirements of the Nagoya Protocol regarding access to genetic resources and the fair and equitable sharing of benefits arising from their utilization [14]. To date, 3 European collections are recognized [15].

In 2015, the Nature Open Library program was launched in order to share this unique PFL ressource with industrial or academic partners and to foster the research, development, and industrialization of plant assets. In this context, PFL provided access to their private plants collection, including some rare species [16]. Upon restructuration, this program was discontinued as of 2018. Some academic collaborations established at this time were kept active. The partnership established between LPBNP and PFL in 2017 had as principal objective the chemical characterization of the full PFL plant extracts collection. In order to evaluate the feasibility of such an ambitious program, a pilot project was defined. It focused on a selection of 1,600 samples (corresponding to approximately 10% of the extracts prepared in the full PFL collection). The results of this pilot study—namely, the generated data and outcomes—are shared in this Datanote.

Note: from now on, in this article, the full collection (17,000 extracts) is referred to as the “PFL collection”. The selected set is simply referred to as “the 1,600 extracts collection”.

### Sample collection

**Figure 1:**
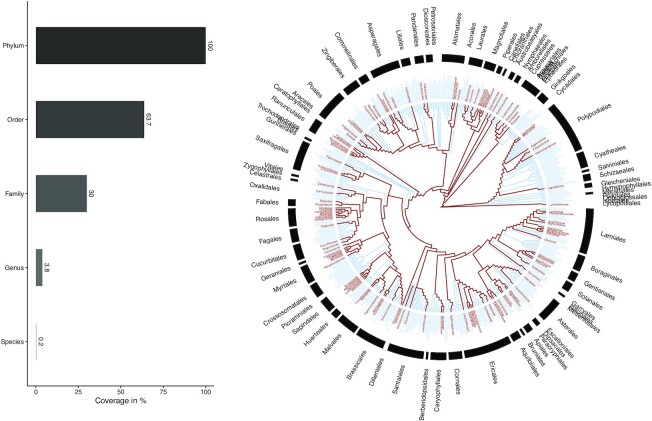
**Taxonomical coverage of the profiled collection (1,600)**. On the left, the barplot represents the overall coverage at main taxa level and up to the phylum Streptophyta. On the right, the taxonomical coverage is represented using a taxonomical tree of all families within the Streptophyta phylum. The families present within the current collection are highlighted in red. The Open Tree of Life (ott3.3) was used for taxonomy resolving. Download pdf version here.

Aa previously detailed, all samples from the PFL collection were collected with respect to the different regulations around genetic resources at the time of collection. After drying and grounding, they were stored at room temperature, in the dark, in high-density polyethylene barcoded 0.5-L or 2L-pots. The storage room is access secured and protected by an automatic fire protection system with inert gas (50% nitrogen, 50% argon). Precise localization of the geographical sampling sites, unique IDs, barcodes, and quantities are stored in the PFL internal data management system. Furthermore, to perform later sample inspections (e.g., in case of dubious botanical identifications), all dry plant parts are also sampled and stored in their intact form. Several constraints were followed while building this collection. First, to have the best chances of finding new chemical entities, a large chemical diversity was desirable. Taking the assumption that taxonomical position and chemical production were related, samples were collected in order to maximize diversity within superior plant taxa (classes, orders, families, and genera; see Fig. 1). Second, only plants providing reasonable biomass quantity (i.e., >50 g dry ground samples) were collected, hence allowing isolation of potentially active compounds in sufficient quantity to be characterized and to perform preliminary bioassays. Third, for preserving samples on the long term but also for allowing only stable compounds to be left in the samples, they were dried for 3 days at 55°C. This point foresaw issues of potential HTS hits due to unstable compounds that would be difficult to isolate and manipulate.

The phylogenetic coverage of the 1,600 extracts collection is depicted in Fig. 1. Within the Streptophyta (Q133527) phylum, the collection represented ca. 64% of the known orders and ca. 30% of all known botanical families. The numbers rapidly decreased to ca. 4% of the known genus and less than 0.5% of all known species. See horizontal barplot in Fig. 1. The botanical families of the collection are relatively well distributed across the global phylogeny, despite some orders (e.g., Sapindales [Q26316], Rosales [Q21895]) being better covered than others (e.g., Crossosomatales [Q21860], Cyatheales [Q623232]). Interactive barplots are available for inspection of the coverage of orders (by families) and of families (by genus). The scripts for taxonomical name resolving and Fig. 1. generation are available online [17].

### Extraction and sample preparation

Since the PFL collection was designed to provide extracts for HTS experiments, medium-polarity compounds were targeted. Using ethyl acetate (EtOAc) extracts purified over silica gel allowed reducing the amount of known classes of pan-assay interference compounds such as condensed tannins [18].

Starting from the dried plant material, the extracts were prepared as follows. A sample of 8 g (leaves, whole plant or aerial parts) or 10 g (subterranean parts, roots, bark) was extracted over a period of 8 to 10 hours with 80 mL or 100 mL EtOAc, respectively. The extracts were filtered through paper and dried under vacuum in a rotary evaporator to a reduced volume (ca. 5 mL). The residue was transferred to a preweighted recipient and dried under vacuum until measurement of constant weight. The extracts were dissolved in EtOAc at a concentration of 30 mg/mL. An aliquot of 1 mL was mixed with 200 mg silica gel (Merk 60, 40–63 μm) and dried under vacuum. The resulting powdery mixture was transferred to a SPE cartridge (6 mL, 1 g SiO_2_). The cartridge was eluted with 10 mL dichloromethane to remove apolar compounds, and then the cartridge was eluted with 8 mL of a mixture dichloromethane/methanol (85:15) and subsequently washed with 2 mL of the same mixture to fully recover the compounds of interest. The filtrate (ca. 10 mL final volume) was dried under vacuum. Extracts were then dissolved in dimethyl sulfoxide (DMSO) to a concentration of 5 mg/mL and transferred to 1-mL 96 deep-well plates for further analysis. For liquid chromatography–mass spectrometry (LC-MS) analysis (see below), the extracts were dissolved in DMSO to reach a concentration of 2.5 mg/mL. The plates originally contained 80 extracts per plate, with columns 1 and 12 being empty. These columns were filled, respectively, by a quality control (QC) sample and a DMSO blank. The QC samples were constituted by a mixture of *Cinchona pubescens* Vahl (Q164574), *Panax ginseng* C.A.Mey. (Q182881), *Ginkgo biloba* L. (Q43284), *Arnica montana* L. (Q207848), and *Salvia officinalis* L. (Q1111359) methanolic extracts dissolved in a 1:1:1:1:1 ratio at 5 mg/mL in DMSO. These blanks and QC samples were injected every 10 samples.

### LC-MS/MS analyses

Chromatographic separation was performed on a Waters Acquity UPLC I-Class system interfaced to a Q-Exactive Focus mass spectrometer (Thermo Scientific, Bremen, Germany), using a heated electrospray ionization (HESI-II) source. Thermo Scientific Xcalibur 3.1 software was used for instrument control. The LC conditions were as follows: column, Waters BEH C18 50 × 2.1 mm, 1.7 μm; mobile phase, (A) water with 0.1% formic acid; (B) acetonitrile with 0.1% formic acid; flow rate, 600 μL·min^−1^; injection volume, 2 μL; and gradient, linear gradient of 5% to 100% B over 7 minutes and isocratic at 100% B for 1 minute. The optimized HESI-II parameters were as follows: source voltage, 3.5 kV (pos); sheath gas flow rate (N2), 55 units; auxiliary gas flow rate, 15 units; spare gas flow rate, 3.0; capillary temperature, 350.00°C; and S-Lens RF Level, 45. The mass analyzer was calibrated using a mixture of caffeine, methionine–arginine–phenylalanine–alanine–acetate, sodium dodecyl sulfate, sodium taurocholate, and Ultramark 1621 in an acetonitrile/methanol/water solution containing 1% formic acid by direct injection. The data-dependent MS/MS events were performed on the 3 most intense ions detected in full-scan MS (Top3 experiment). The MS/MS isolation window width was 1 Da, and the stepped normalized collision energy was set to 15, 30, and 45 units. In data-dependent MS/MS experiments, full scans were acquired at a resolution of 35,000 full width at half maximum (FWHM) (at *m/z* 200) and MS/MS scans at 17,500 FWHM both with an automatically determined maximum injection time. After being acquired in an MS/MS scan, parent ions were placed in a dynamic exclusion list for 2.0 seconds.

### Data processing and molecular networking

The MS data were converted from .RAW (Thermo) standard data format to .mzXML format using the msconvert module, part of ProteoWizard (RRID:SCR_012056) [19]. The converted files were treated using the MZmine (RRID:SCR_012040) software suite v. 2.53 [20]. The parameters were adjusted as follows: the centroid mass detector was used for mass detection with the noise level set to 1.0E4 for MS level set to 1 and to 0 for MS level set to 2. The ADAP chromatogram builder was used and set to a minimum group size of scans of 5, minimum group intensity threshold of 1.0E4, minimum highest intensity of 5.0E5, and *m/z* tolerance of 12 ppm [21]. For chromatogram deconvolution, the algorithm used was the wavelets (ADAP). The intensity window S/N was used as the S/N estimator, with a signal to noise ratio set at 10, a minimum feature height at 5.0E5, a coefficient area threshold at 130, a peak duration ranges from 0.0 to 0.5 minutes, and the retention time (RT) wavelet range from 0.01 to 0.03 minutes. Isotopes were detected using the isotopes peaks grouper with an *m/z* tolerance of 12 ppm, a RT tolerance of 0.01 minutes (absolute), and the maximum charge set at 2, and the representative isotope used was the most intense. Each feature list was filtered before alignment to keep only features with an associated MS2 scan and an RT between 0.5 and 8.0 minutes using the feature filtering. Peak alignment was performed using the join aligner method (*m/z* tolerance at 40 ppm), absolute RT tolerance at 0.2 minutes, weight for *m/z* at 2, and weight for RT at 1 and a weighted dot-product cosine similarity of 0.3. The aligned feature list (119,182 features) was exported using the export to GNPS module. Features occurring in QC samples only or in blanks were removed before molecular networking, resulting in a final feature list of 117,005 features. The MZmine parameters used for the data treatment are available at the referenced link [22].

The initial study of this aligned peak list with classical unsupervised statistics (e.g., principal component analysis, principal coordinates analysis) indicated a strong batch effect that could be tracked down to a specific date and attributed to a change of column during the course of the mass spectrometry analysis of the full collection. Such batch effects are almost inevitable when samples are profiled on long periods (here spanning over several months) and are particularly complicated to mitigate especially when dealing with chemodiverse datasets where poor overlap among the samples is expected. This incentivized us to develop a novel computational mass spectrometry solution for MS2 BasEd SaMple VectOrization (MEMO - [biotools:memo-ms]). MEMO allows organizing samples in large and chemodiverse collections in a retention-time agonist fashion, thus strongly mitigating the batch effect and allowing the comparison of samples acquired over heterogeneous chromatographic conditions. Applied to the current plant extract collection, MEMO allows efficient reduction of the observable batch effect and clustering samples according to their content [23]. See the referenced link for a view of the batch effect on the classically aligned dataset and its mitigation using MEMO fingerprints [24].

To analyze the spectral diversity of the profile collection, a molecular network (MN) was created on the GNPS (RRID:SCR_019012) website [25] using the .mgf spectra file generated at the previous step [26]. The precursor ion mass tolerance was set to 0.02 Da and an MS/MS fragment ion tolerance of 0.02 Da. A network was then created where edges were filtered to have a cosine score above 0.7 and more than 6 matched peaks. Further, edges between 2 nodes were kept in the network if and only if each of the nodes appeared in each other's respective top 10 most similar nodes. Finally, the maximum size of a spectral family was set to 100, and the lowest scoring edges were removed from molecular families until the molecular family size was below this threshold. The spectra in the network were then searched against GNPS spectral libraries. All matches kept between network spectra and library spectra were required to have a score above 0.7 and at least 6 matched peaks. The molecular networking job results are available online [27]. A Cytoscape (RRID:SCR_003032) file corresponding to the full molecular network mapped with a color layout corresponding to NP Classifier chemical classification [28], the experimental and theoretical spectral matches (see “Metabolite annotation” section for details), and a feature table grouped at the family level are available through the following MassIVE (RRID:SCR_013665) repository link.

Via the GNPS Explorer interface, it is possible to efficiently navigate through the uploaded spectral file and its associated metadata, see referenced link [29]. For example, the name of a plant of interest can be typed under the NCBI Taxonomy header, resulting in the direct filtering of the dataset for this specific plant. Individual spectral files can then be selected (and eventually compared if multiple are selected) and viewed using the GNPS dashboard. See, for example, this interactive view of the total ion chromatogram of *Desmodium heterophyllum* Hook. & Arn (Q10770714) aerial parts.

**Figure 2:**
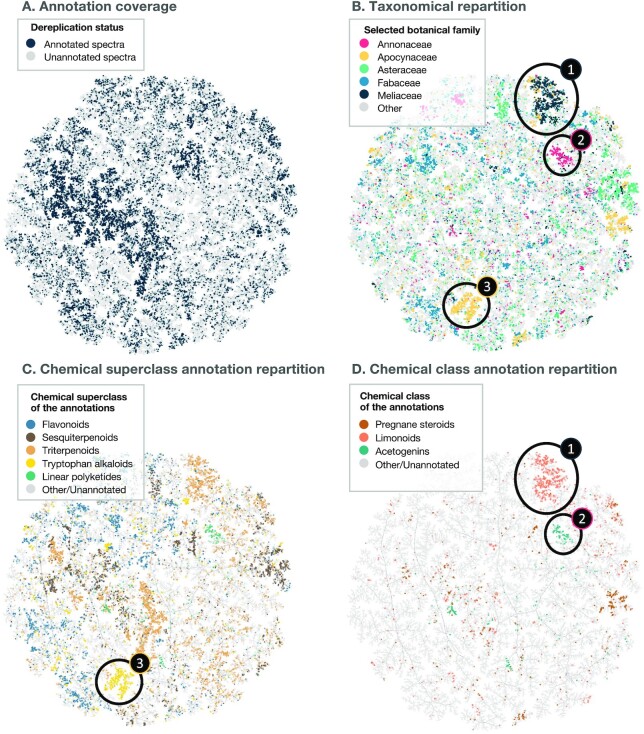
**Spectral diversity of the profiled plant collection (1,600 extracts)**. The TMAP approach is employed to display the >100,000 spectra resulting from the alignment of the 1,600 samples untargeted MS/MS profiles. In this TMAP, each dot represents a feature's spectrum, and they are linked together according to their similarity. In A, blue dots (36% of the total amount of spectra) correspond to annotated spectra while gray dots (64%) correspond to unannotated spectra. In B, dots are colored according to the botanical family of the sample where the highest MS1 peak area for the corresponding feature was recorded. In C and D, dots are colored according to the NPClassifier superclass and class, respectively, of their annotation (for annotated dots). In B, it is possible to spot spectral regions (1, 2, and 3) specific to given botanical families. Region 1 is specific to the Meliaceae family, and these spectra are mainly annotated as limonoid derivatives, region 2 is specific to the Annonaceae family and spectra are mainly annotated as acetogenin derivatives, and region 3 is specific to the Apocynaceae family and spectra are annotated as tryptophan alkaloid derivatives. Note that the structural annotation results are reweighed according to the taxonomical proximity of the biological source of the candidate structure and the biological source of the annotated spectra. A bias favoring taxa-specific structures can thus be observed. This interactive structural TMAP can be browsed online [34].

In addition to the MN, spectra (from the same .mgf file used for MN) were organized using TMAP visualization [30]. In this case, the TMAP corresponds to a minimum spanning tree built from a dense network of spectral similarity. For the establishment of this visualization, spectra were first translated to documents (2 decimals were used, i.e., a peak at 100.3897 would be translated as “peak@100.39”) using matchms (biotools:matchms) and spec2vec (biotools:spec2vec) packages [31, 32], with calculation of neutral losses (up to 400 *m/z*) to the precursor. The spectral documents (i.e., a list of peaks and losses without intensity information) were then hashed using the MinHash scheme and indexed in an locality-sensitive hashing (LSH) forest that was used to generate the TMAP visualization based on the presence/absence of peaks and losses in spectra [30, 33]. The generated TMAP is presented in Fig. 2, an interactive visualization is available [34], and the code used for the TMAP generation is available online [17]. Such visualization allows to highlight taxa-specific spectral subspaces. In addition, structural annotation (see next section for details concerning the metabolite annotation process) were overlaid to their corresponding spectra. When no annotations were reported, a NanoPutian was depicted as a placeholder. The structural annotations were limited to compounds reported in the Eukaryote domain. For example, the spectra region 1 in Fig. 2B and 2D is mainly specific to samples belonging to the Meliaceae (Q158979) family, and most of these spectra are annotated as limonoid derivatives (Q669514). Region 2 in Fig. 2, contains mainly spectra specific to the Annonaceae (Q220025) family and corresponds to acetogenin derivatives (Q3604300). Finally, the spectral region 3 is specific to the Apocynaceae family (Q173756) and is mainly annotated as tryptophan alkaloid derivatives. On the other hand, botanical families such as the Fabaceae (Q44448), light blue in Fig. 2B, appear to occupy a much wider spectral space. These examples showcase the importance of the taxonomic coverage of an extract collection to maximize the chemical diversity. Indeed, if some chemical classes, such as the flavonoid (Q3561192) derivatives, blue in Fig. 2C, appear to be widespread, some others are known to be restricted to specific taxa, such as the acetogenins to the Annonaceae [35]. The interpretation of this spectral TMAP is highly dependent on the structural annotations’ quality (see next section “Metabolite annotation”) and as such should be taken with caution. In particular, because the annotation process favored structures reported in closer taxa, it should also be noted that the comparison of annotation occurrences and taxonomy may be impacted by an annotation set biased toward denser taxa-specific ensembles of structures.

### Metabolite annotation

#### Experimental spectral libraries search

The full spectral dataset corresponding to the 1,600 plant extract collection has been uploaded on the MassIVE repository [36], and a continuous identification workflow is automatically carried against GNPS experimental spectral libraries. The latest results of this continuous identification workflow can be observed online [37]. The latest iteration at the time of writing (18 April 2022) indicated that a total of 2,665 unique compounds were spectrally matched [38].

#### Theoretical spectral libraries search

In addition to an experimental spectral library search, we have shown that spectral matching against theoretical spectral libraries of natural products was an efficient way to cover a much wider, yet relevant, spectral space [39]. Furthermore, we showed that taking into account the taxonomical distance between the biological source of candidate structure and the biological source of the annotated extracts greatly improved the overall quality of the annotation results [40]. Thus, in addition to the spectral search performed at the molecular networking step against publicly available spectral libraries (see previous section), a taxonomically informed metabolite annotation was performed. For this, we first established a large theoretical spectral database of natural products following a previously established methodology [39]. This spectral database and associated biological sources metadata were constructed using chemical structure and information compiled during the LOTUS Initiative's first project (biotools:lotus-db) aiming to establish an open and evolutive resource compiling natural product biological occurrences [41]. The theoretical spectral database is publicly available [42]. The biological sources metadata are available online [43]. The taxonomically informed metabolite annotation was performed using the met_annot_enhancer scripts [44]. The parameters used for the taxonomically informed metabolite annotation process and the resulting tables can be found online [45]. PF_full_datanote_spectral_match_results_repond.tsv corresponds to the Cytoscape formatted output.

#### Visualization of the metabolite annotations

**Figure 3:**
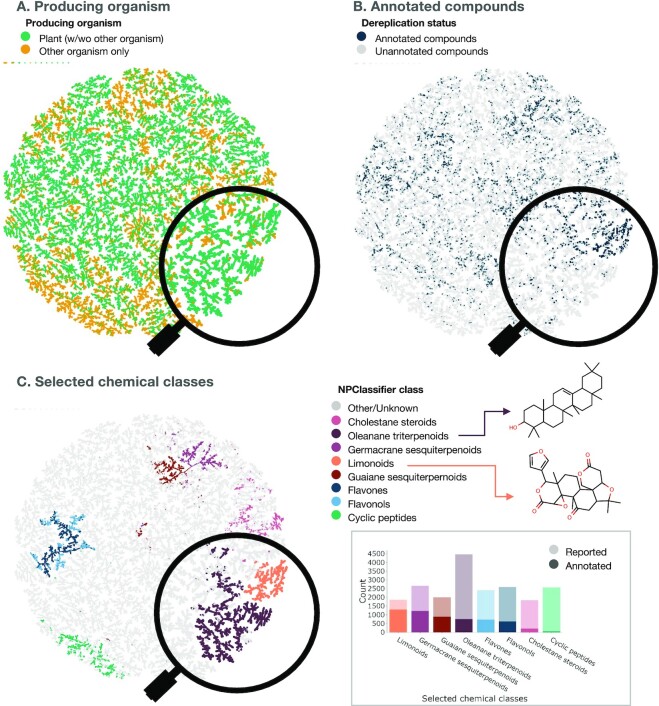
**Chemical diversity of the profiled plant collection (1,600 extracts) and coverage against reported natural products**. Visualization of reported natural products structures (LOTUS v1 and Dictionary of Natural Products v29.1) as a TMAP with plotting of the producing organism (A), the annotation's status (i.e., whether the 2-dimensional structure was annotated in the dataset) (B), and selected NPClassifier classes (C). In the insert of C, the barplot represents the number of compounds reported for each of the selected chemical classes with the opaque part of the bar representing the annotated compounds. The zoom on the limonoid and oleanane terpenoid clusters of the TMAP allows visualizing a well-covered chemical class such as limonoids and a less-covered one such as oleanane triterpenoids. A specific member of each class (limonin [Q2398745] for the limonoids and β-amyrin [Q27108621] for the oleanane triterpenoids) is represented in their planar structure form for illustration purposes. This interactive structural TMAP can be browsed online [51].

In order to obtain an overview of the metabolite annotation results on the profiled collection, these were compared to the ensemble of molecules described in LOTUS (see Fig. 3). For this, a TMAP [30, 46, 47] was built to connect similar chemical structures using the MAP4 (MinHashed Atom-Pair fingerprint up to 4 bonds) fingerprint [48], and the retrieval of the nearest neighbors was achieved as described above for the spectral TMAP construction. In Fig. 3, each dot represents a chemical structure and is linked to its neighbor according to its structural proximity. In Fig. 3A, the color code indicates the repartition of chemical structures mostly found to be present at least once in plants (green color) versus structures found only in other kingdoms (orange color). In Fig. 3B, each of the annotated structures within the 1,600 plant extract collection is displayed (strong blue) within the rest of the reported structures in LOTUS (light gray) indicating a relatively heterogeneous structural coverage of the annotations with some denser areas. Finally, the color coding in Fig. 3C allows one to distinguish chemical classes in the overall TMAP. The NPClassifier classification is employed [49]. The framed barplot displays the most frequently annotated chemical classes in the present dataset (strong color) versus the total count for each class in LOTUS (light color). Multiple factors can explain discrepancies between the repartition of chemical classes annotated in the 1,600 extracts collection versus the overall repetition observed in LOTUS. For example, cyclic peptides (green bar in the framed barplot) are poorly covered. This can be explained by the fact that such structures are most often found in microbial organisms (as observed when checking this region in plot A). On the other hand, the high coverage of limonoids in the collection can be explained by the high proportion of species from the Sapindales order (see Fig. 1 and the orders coverage interactive barplot) and the fact that this order is known to be mainly responsible for the biosynthesis of such scaffolds [50]. An interactive version of the structural TMAP presented in Fig. 3 is available online [51].

A detailed coverage of annotations of the dataset versus the full LOTUS annotations is available as interactive plots (coverage at the pathway level, at the superclass level, and at the class level.)

## Reusability

Regarding data reuse, multiple options exist for such a dataset. Hereafter, we describe some selected use cases. For example, GNPS continuous identification can be explored and retrieved online [52] and can be used to compare or combine the results of experimental library matches to theoretical spectral library matches. Since the dataset is also available through the ReDu interface, multiple reanalysis possibilities can be explored. For example, through the https://redu.ucsd.edu/metadataselection [53] interface and after selection of the PF dataset by its DatasetAccession value (MSV000087728), a filter for all files of the dataset can be established. Filters of individual species can be obtained by selecting their corresponding NCBITaxonomy terms. Later on, the full set or subset thereof can be selected for Molecular Networking or Library Search at GNPS, for example. See the complete ReDu documentation for details [54].

The recently developed Mass Spec Query Language (MassQL) allows efficient search for specific patterns within large spectral datasets [55]. For example, researchers can look for ionization patterns characteristic of dimeric monoterpene indole alkaloids (MIAs) using the following MassQL query. The results of the query on the 1,600 extracts collection is available here. As an example, this query returns that a feature with a mass of 687.3570 corresponds to the searched criteria. This page displays the overlap of the MassQL query criteria and the corresponding MS1 and MS2 spectra, and it also indicates that these spectra were found in sample VGF152_B02_pos.mzXML. We can then turn to the GNPS Explorer interface for the 1,600 plants dataset, filter for the sample ID, and see that this sample is an extract of *Tabernaemontana coffeoides* (Q15376858), which is known to contain dimeric MIA. In the GNPS Explorer interface, we can quickly filter for other species of *Tabernaemontana* (one is present, *Tabernaemontana crassa* [Q14862275]) and use the GNPS LCMS Dashboard (see plots) to observe that the incriminated feature is mostly present in *T. coffeoides*. A MassQL compendium groups query examples, and these can be used to further explore the 1,600 extracts collection.

## Conclusions

We presented the results of state-of-the-art computational approaches used for both spectral organization and metabolite annotation of high-resolution mass spectrometry data acquired on a large collection of 1,600 chemically diverse plant extracts. All original mass spectrometry profiles and associated metadata are made available to the community for further (re)analysis. The results of the metabolite annotation define a putative chemical space that can be exploited and refined in subsequent drug discovery, chemotaxonomic, or ecometabolomics projects. Here, we illustrate that, through partnerships between academia and industry, the faith of historical and private collections of plant extracts can be changed and the richness of the associated chemical diversity can be made available to a wider public.

## Availability of Source Code and Requirements

The scripts used to generates the figures of this Data Note are available as follows:

Project name: Scripts for the PF1600 project

Project homepage: https://github.com/mandelbrot-project/pf_1600_datanote

Operating system: Platform independent

Programming language: R (RRID:SCR_001905) and Python (RRID:SCR_008394)

License: GNU Affero General Public License v3.0

## Data Availability

All .RAW (Thermo), .mzXML, and .mzML datafiles in positive and negative ionization modes along with metadata and metabolite annotation tables are available on the MassIVE repository under accession number MSV000087728 [36]. Molecular networking job results are publicly available [56]. Furthermore, all supporting data and materials are also available in the *GigaScience* GigaDB database [57].

## Abbreviations

DMSO: dimethyl sulfoxide; EtOAc: ethyl acetate; FWHM: full width at half maximum; HESI-II: heated electrospray ionization; HTS: high-throughput screening; LC-MS: liquid chromatography–mass spectrometry; LPBNP: Laboratory of Phytochemistry and Bioactive Natural Products; LSH: locality-sensitive hashing; MAP4: MinHashed Atom-Pair fingerprint up to 4 bonds; MassQL: Mass Spec Query Language; MEMO: MS2 BasEd SaMple VectOrization; MN: molecular network; PFL: Pierre Fabre Laboratories; QC: quality control; TMAP: Tree MAP; UMAP: uniform manifold approximation and projection.

## Competing Interests

The authors declare that they have no competing interests.

## Funding

Supported by the Swiss National Science Foundation (SNF N°CRSII5_189921/1) to J-L.W, A.R. and P-M.A.

## Authors' Contributions

Conceptualization: P.-M.A., B.D., and J.-L.W. Data curation: P.-M.A., A.Ga., L.Q.G., A.R., and M.D.K. Funding acquisition: P.-M.A. and J.-L.W. Investigation: P.-M.A. and M.D.K. Methodology: P.-M.A., M.D.K., and J.-L.W. Project administration: P.-M.A., B.D., and J.-L.W. Resources: C.L., A.Gr., B.D., and J.-L.W. Software: P.-M.A., A.Ga., T.W., E.D., and A.R. Supervision: P.-M.A. and J.-L.W. Validation: P.-M.A., A.Ga., and M.D.K. Visualization: P.-M.A., A.Ga., E.D., and A.R. Writing—original draft: P.-M.A. Writing—review and editing: P.-M.A., A.Ga., L.Q.G., A.R., and A.Gr.

## Supplementary Material

giac124_Authors_Response_To_Reviewer_Comments

giac124_GIGA-D-22-00126_Original_Submission

giac124_GIGA-D-22-00126_Revision_1

giac124_GIGA-D-22-00126_Revision_2

giac124_Response_to_Reviewer_Comments_Original_Submission

giac124_Reviewer_1_Report_Original_Submission Kyo Bin Kang -- 6/17/2022 Reviewed

giac124_Reviewer_1_Report_Revision_1 Kyo Bin Kang -- 9/19/2022 Reviewed

giac124_Reviewer_2_Report_Original_Submission Mingxun Wang -- 6/23/2022 Reviewed

## References

[1] Duflos A, Kruczynski A, Barret J-M. Novel aspects of natural and modified Vinca alkaloids. Curr Med Chem Anticancer Agents. 2002;2:55–70. 12678751 10.2174/1568011023354452

[2] Fiorini-Puybaret C, Joulia P. Dye composition comprising a combination of two plant extracts of *Lawsonia inermis*. World Patent WO2020249748A1.

[3] Nguyen T, Cousy A, Steward N. Method for producing celastrol and pentacyclic triterpene derivatives. World Patent WO2017194757A1.

[4] Vandenberghe I, Créancier L, Vispé S, et al. Physalin B, a novel inhibitor of the ubiquitin-proteasome pathway, triggers NOXA-associated apoptosis. Biochem Pharmacol. 2008;76:453–62. 18577376 10.1016/j.bcp.2008.05.031

[5] Pouny I, Long C, Batut M, et al. Quinolizidine alkaloids from *Cylicomorpha solmsii*. J Nat Prod. 2021;84:1198–202. 33606529 10.1021/acs.jnatprod.0c01261

[6] Long C, Sauleau P, David B, et al. Bioactive flavonoids of *Tanacetum parthenium* revisited. Phytochemistry. 2003;64:567–9. 12943776 10.1016/s0031-9422(03)00208-5

[7] Pouny I, Batut M, Vendier L, et al. Cytisine-like alkaloids from *Ormosia hosiei* Hemsl. & E.H. Wilson. Phytochemistry. 2014;107:97–101. 25172516 10.1016/j.phytochem.2014.07.022

[8] Begue J-P, Bonnet-Delpon D, Crousse B, et al. Dimeric derivatives of artemisinin and application in anti-cancer therapy. World Patent WO2010012761A1.

[9] Marion F, Kaloun EB, Lieby-Muller F, et al. Flavagline derivatives. World Patent WO2016001441A1.

[10] Marion F, Lieby-Muller F, Grisoni S, et al. Griseofulvin derivatives. World Patent WO2014020101A1.

[11] Marion F, Annereau J-P, Fahy J. Nitrogenated derivatives of pancratistatin. World Patent WO2010012714A1.

[12] Beck J, Guminski Y, Long C, et al. Semisynthetic neoboutomellerone derivatives as ubiquitin-proteasome pathway inhibitors. Bioorg Med Chem. 2012;20:819–31. 22206869 10.1016/j.bmc.2011.11.066

[13] Kaloun EB, Long C, Molinier N, et al. Partial synthesis of 14-deoxy-14-aminotriptolide. Tetrahedron Lett. 2016;57:1895–8.

[14] EUR-Lex—32014R0511—EN—EUR-Lex. http://data.europa.eu/eli/reg/2014/511/oj. Accessed 2022 Nov 12.

[15] Register of Collections—EU ABS Regulation. https://ec.europa.eu/environment/nature/biodiversity/international/abs/pdf/Register%20of%20Collections.pdf. Accessed 2022 Nov 11.

[16] Pierre Fabre laboratories unveils Open Nature Library, a unique open innovation program in the world. https://www.pierre-fabre.com/en/press_release/pierre-fabre-laboratories-unveils-open-nature-library-a-unique-open-innovation. Accessed 2022 Nov 11.

[17] pf_1600_datanote scripts. GitHub. https://github.com/mandelbrot-project/pf_1600_datanote/releases/tag/v0.1. Accessed 2022 Nov 11.

[18] David B, Ausseil F. High throughput screening of vegetal natural substances. In: Hostettmann K, ed. ​​​​​Handbook of Chemical and Biological Plant Analytical Methods. Chichester, UK: John Wiley & Sons, Ltd; 2014:887–1010.. 10.1002/9780470027318.a9944.

[19] Chambers MC, Maclean B, Burke R, et al. A cross-platform toolkit for mass spectrometry and proteomics. Nat Biotechnol. 2012;30:918–20. 23051804 10.1038/nbt.2377 PMC3471674

[20] Pluskal T, Castillo S, Villar-Briones A, et al. MZmine 2: Modular framework for processing, visualizing, and analyzing mass spectrometry-based molecular profile data. BMC Bioinf. 2010;11:395. 10.1186/1471-2105-11-395 PMC2918584 20650010

[21] Myers OD, Sumner SJ, Li S, et al. One step forward for reducing false positive and false negative compound identifications from mass spectrometry metabolomics data: new algorithms for constructing extracted ion chromatograms and detecting chromatographic peaks. Anal Chem. 2017;89:8696–703. 28752754 10.1021/acs.analchem.7b00947

[22] 210302_VGF_pos_parameters.xml. https://massive.ucsd.edu/ProteoSAFe/DownloadResultFile?file=f.MSV000087728/updates/2022-05-02_pmallard_e88304cd/other/210302_VGF_pos_parameters.xml. Accessed 2022 Nov 11.

[23] Gaudry A, Huber F, Nothias L-F, et al. MEMO: mass spectrometry-based sample vectorization to explore chemodiverse datasets. Front Bioinform. 2022;2:842964. 10.3389/fbinf.2022.842964 PMC9580960 36304329

[24] Comparative PCoA with samples colored according to their injection date (2 groups). https://mandelbrot-project.github.io/memo_publication_examples/plant_extract_dataset/pcoa_vgf_color_before_after.html. Accessed 2022 Nov 11.

[25] GNPS. http://gnps.ucsd.edu. Accessed 2022 Nov 11.

[26] Wang M, Carver JJ, Phelan VV, et al. Sharing and community curation of mass spectrometry data with GNPS. Nat Biotechnol. 2016;34:828–37. 27504778 10.1038/nbt.3597 PMC5321674

[27] UCSD Computational Mass Spectrometry Website. https://proteomics2.ucsd.edu/ProteoSAFe/status.jsp?task=3197f70bed224f9ba6f59f62906839e9. Accessed 2022 Nov 11.

[28] Kim HW, Wang M, Leber CA et al. NPClassifier: A deep neural network-based structural classification tool for natural products. J Nat Prod. 2021;84(11):2795–807. 34662515 10.1021/acs.jnatprod.1c00399 PMC8631337

[29] GNPS—Dataset Browser. https://gnps-explorer.ucsd.edu/MSV000087728?dataset_accession=MSV000087728&metadata_source=MASSIVE&metadata_option=f.MSV000087728%2Fupdates%2F2021-11-15_pmallard_334e9199%2Fmetadata%2Fgnps_metadata.tsv. Accessed 2022 Nov 11.

[30] Probst D, Reymond J-L. Visualization of very large high-dimensional data sets as minimum spanning trees. J Cheminform. 2020;12:12. 33431043 10.1186/s13321-020-0416-x PMC7015965

[31] Huber F, Verhoeven S, Meijer C, et al. Matchms—processing and similarity evaluation of mass spectrometry data. J Open Source Softw. 2020;5:2411.

[32] Huber F, Ridder L, Verhoeven S, et al. Spec2Vec: Improved mass spectral similarity scoring through learning of structural relationships. PLoS Comput Biol. 2021;17:e1008724. 33591968 10.1371/journal.pcbi.1008724 PMC7909622

[33] LSH forest: self-tuning indexes for similarity search. ACM Digital Library. 10.1145/1060745.1060840. Accessed 2022 Nov 12.

[34] pf1600_spectral_tmap_pos. https://mandelbrot-project.github.io/pf_1600_datanote/data/outputs/spectral/tmap/pf1600_spectral_tmap_pos.html. Accessed 2022 Nov 11.

[35] Neske A, Ruiz Hidalgo J, Cabedo N, et al. Acetogenins from Annonaceae family. Their potential biological applications. Phytochemistry. 2020;174:112332. 32200068 10.1016/j.phytochem.2020.112332

[36] Allard P-M, Wolfender J-L. MassIVE MSV000087728 - GNPS_PF_plant_extracts_library_dataset_01. MassIVE 2021. 10.25345/c59j97. Accessed 2022 Nov 11.

[37] UCSD Computational Mass Spectrometry Website. https://gnps.ucsd.edu/ProteoSAFe/result.jsp?task=b753bf1e39cb4875bdf3b786e747bc15&view=advanced_view. Accessed 2022 Nov 11.

[38] UCSD Computational Mass Spectrometry Website. https://gnps.ucsd.edu/ProteoSAFe/result.jsp?task=ee2e8e9cf8214f48ab6ee01df652a3f2&view=all_unique_compounds. Accessed 2022 Nov 11.

[39] Allard P-M, Péresse T, Bisson J, et al. Integration of molecular networking and in-silico MS/MS fragmentation for natural products dereplication. Anal Chem. 2016;88:3317–23. 26882108 10.1021/acs.analchem.5b04804

[40] Rutz A, Dounoue-Kubo M, Ollivier S, et al. Taxonomically informed scoring enhances confidence in natural products annotation. Front Plant Sci. 2019;10:1329. 31708947 10.3389/fpls.2019.01329 PMC6824209

[41] Rutz A, Sorokina M, Galgonek J, et al. The LOTUS initiative for open knowledge management in natural products research. eLife. 2022;11:e70780. 10.7554/eLife.70780 PMC9135406 35616633

[42] Allard P-M, Bisson J, Rutz A. ISDB: In Silico Spectral Databases of Natural Products. Zenodo 2021. 10.5281/zenodo.5607264.

[43] Rutz A, Bisson J, Allard P-M. The LOTUS Initiative for Open Natural Products Research: frozen dataset union wikidata (with metadata). Zenodo 2022. 10.5281/zenodo.6378204.

[44] met_annot_enhancer v0.1. GitHub. https://github.com/mandelbrot-project/met_annot_enhancer/releases/tag/v0.1 Accessed 2022 Nov 11.

[45] MassIVE Dataset Files. https://massive.ucsd.edu/ProteoSAFe/dataset_files.jsp?task=b753bf1e39cb4875bdf3b786e747bc15#%7B%22table_sort_history%22%3A%22main.collection_asc%22%2C%22main.attachment_input%22%3A%22updates%2F2022-04-28_pmallard_b0e0f70c%22%7D. Accessed 2022 Nov 11.

[46] Probst D, Reymond J-L. SmilesDrawer: parsing and drawing SMILES-encoded molecular structures using client-side JavaScript. J Chem Inf Model. 2018;58:1–7. 29257869 10.1021/acs.jcim.7b00425

[47] Probst D, Reymond J-L. FUn: a framework for interactive visualizations of large, high-dimensional datasets on the web. Bioinformatics. 2018;34:1433–5. 29186333 10.1093/bioinformatics/btx760

[48] Capecchi A, Probst D, Reymond J-L. One molecular fingerprint to rule them all: drugs, biomolecules, and the metabolome. J Cheminform. 2020;12:43. 33431010 10.1186/s13321-020-00445-4 PMC7291580

[49] Kim HW, Wang M, Leber CA, et al. NPClassifier: a deep neural network-based structural classification tool for natural products. J Nat Prod. 2021;84:2795–807. 34662515 10.1021/acs.jnatprod.1c00399 PMC8631337

[50] Hodgson H, De La Peña R, Stephenson MJ, et al. Identification of key enzymes responsible for protolimonoid biosynthesis in plants: Opening the door to azadirachtin production. Proc Natl Acad Sci. 2019;116:17096–104. 31371503 10.1073/pnas.1906083116 PMC6708365

[51] pf1600_structural_tmap. https://mandelbrot-project.github.io/pf_1600_datanote/data/outputs/structural/tmap/pf1600_structural_tmap.html. Accessed 2022 Nov 11.

[52] UCSD Computational Mass Spectrometry Website. https://gnps.ucsd.edu/ProteoSAFe/status.jsp?task=ee2e8e9cf8214f48ab6ee01df652a3f2. Accessed 2022 Nov 11.

[53] ReDU. https://redu.ucsd.edu/metadataselection. Accessed 2022 Nov 11.

[54] ReDU Documentation. https://mwang87.github.io/ReDU-MS2-Documentation. Accessed 2022 Nov 11.

[55] Jarmusch AK, Aron AT, Petras D, et al. A universal language for finding mass spectrometry data patterns. bioRxiv. 2022.2022.08.06.503000. 10.1038/s41592-025-02660-z PMC12334354 40355727

[56] UCSD Computational Mass Spectrometry Website. https://proteomics2.ucsd.edu/ProteoSAFe/status.jsp?task=3197f70bed224f9ba6f59f62906839e9. Accessed 2022 Nov 11.

[57] Allard P-M, Gaudry A, Quirós-Guerrero L, et al. Supporting data for “Open and re-usable annotated mass spectrometry dataset of a chemodiverse collection of 1,600 plant extracts.” GigaScience Database. 2022. 10.5524/102323. PMC9845059 36649739

