# Supplementary material for: Open and reusable annotated mass spectrometry dataset of a chemodiverse collection of 1,600 plant extracts
Source: Gigascience. 2023 Jan 18;12:giac124. doi: 10.1093/gigascience/giac124 (PMC9845059; doi:10.1093/gigascience/giac124)

GigaScience

Open and re-usable annotated mass spectrometry dataset of a chemodiverse collection of 1,600 plant extracts.

--Manuscript Draft--

**Manuscript Number:**	GIGA-D-22-00126R1
**Full Title:**	Open and re-usable annotated mass spectrometry dataset of a chemodiverse collection of 1,600 plant extracts.
**Article Type:**	Data Note
**Funding Information:**	Schweizerischer Nationalfonds zur Förderung der Wissenschaftlichen Forschung (CRSII5_189921/1) — Pr. Jean-Luc Wolfender
**Abstract:**	As privileged structures, natural products often display potent biological activities. However, the discovery of novel bioactive scaffolds is often hampered by the chemical complexity of the biological matrices they are found in. Large natural extracts collections are thus extremely valuable for their chemical novelty potential but also complicated to exploit in the frame of drug-discovery projects. In the end, it is the pure chemical substances that are desired for structural determination purposes and bioactivity evaluation. Researchers interested in the exploration of large and chemodiverse extracts collections should thus establish strategies aiming to efficiently tackle such chemical complexity and access these structures. Establishing carefully crafted digital layers documenting the spectral and chemical complexity as well as bioactivity results of natural products extracts collections can help to prioritize time-consuming but mandatory isolation efforts. In this note, we report the results of our initial exploration of a collection of 1,600 plant extracts in the frame of a drug discovery effort. After describing the taxonomic coverage of this collection, we present the results of its liquid chromatography high-resolution mass spectrometric profiling and the exploitation of these profiles using computational solutions. The resulting annotated mass spectral dataset and associated chemical and taxonomic metadata are made available to the community and data reuse cases are proposed. We are currently continuing our exploration of this plant extracts collection for drug-discovery purposes (notably looking for novel anti-trypanosomatids, anti-infective and prometabolic compounds) and eco-metabolomics insights. We believe that such a dataset can be exploited and reused by researchers interested in computational natural products exploration.
**Corresponding Author:**	Pierre-Marie Allard<br>University of Fribourg<br>Fribourg, Freiburg SWITZERLAND
**Corresponding Author Secondary Information:**	
**Corresponding Author's Institution:**	University of Fribourg
**Corresponding Author's Secondary Institution:**	
**First Author:**	Pierre-Marie Allard
**First Author Secondary Information:**	
**Order of Authors:**	Pierre-Marie Allard
	Arnaud Gaudry
	Luis-Manuel Quirós-Guerrero
	Adriano Rutz
	Miwa Dounoue-Kubo
	Tom W. N. Walker

Powered by Editorial Manager® and ProduXion Manager® from Aries Systems Corporation

	Emmanuel Defossez
	Christophe Long
	Antonio Grondin
	Bruno David
	Jean-Luc Wolfender
**Order of Authors Secondary Information:**	
**Response to Reviewers:**	Reviewer reports:<br><br>Reviewer #1: This Data Note describes a LC-MS/MS dataset acquired from 1,600 plant extracts, which are a part of the Pierre Fabre extract collection. The entire data is available through MassIVE, a proper public repository for LC-MS/MS-based metabolomics dataset, with an organized metadata. The background history and technical method are well described. All the curated data are accessible through hyperlinks. Large-scale mass spectrometry data on plant specialized metabolites cannot be found commonly, so this Data Note have its own value, and it will be beneficial to the other researchers in relevant fields. Thus, I recommend the publication of this Data Note.<br>I only have minor comments as below:<br><br>- Abstract should clarify that this dataset came from LC-MS/MS.<br><br>Done in “After describing the taxonomic coverage of this collection, we present the results of its liquid chromatography high-resolution mass spectrometric profiling and the exploitation of these profiles using computational solutions.” and keyword LCMS<br><br>- If the authors can give some examples how (I mean technically) this dataset could be re-used, it will be beneficial to the readers. Recently some softwares enhancing data re-use in metabolomics have launched, so maybe they are worth to be mentioned.<br><br>We thank the reviewer for this suggestion. To exemplify and showcase reuse possibilities we moved the Data Availability section to the main text, renamed it Data availability and reusability and presented a series of advanced reused possibilities using notably, ReDu and MassQL. See main text.<br><br>- The authors used the word 'exploitation' several times, but it is not clear what it means exactly. Please replace it with a word describing what was exactly provided from the data curation.<br><br>We changed two occurrences of exploitation for exploration. We employed exploitation in the sense that data can be seen as a resource and thus be exploited in several manners.<br><br>- Introduction: As a researcher outside Europe, I cannot understand what the fact that the PFL collection was 'registered' at the European Commission means; registered for what, or on which list? Please specify this.<br><br>We thank the reviewer for this comment and clarified the implication accordingly in the main text. See “The PFL collection, which is among the largest collection plant samples worldwide with over 17,000 unique samples, was registered on April 1, 2020 at the European Commission under the accession number 03-FR-2020. This official registration recognizes the legality of the access and management process. In detail it means that the collection meets the criteria set out in the EU ABS Regulation which implements at the European level the requirements of the Nagoya Protocol regarding access to genetic resources and the fair and equitable sharing of benefits arising from their utilization. [14] To date three european collections are recognized (https://ec.europa.eu/environment/nature/biodiversity/international/abs/pdf/Register%20of%20Collections.pdf).”<br><br><br>- In discussion on the taxonomic coverage, the absolute size of each taxon (order and family) should be considered. E.g. In the World Flora Online, 5615 species are enlisted

in Sapindales while 481 species in Cyatheales.

Following this comment and to further document the coverage we prepared two interactive plots available for the readers to explore 1) the coverage according to the numbers of families per order 2) the coverage according to the numbers of genus per family. See also the main text : “Interactive bar plots are available for the inspection of the coverage of orders (by families) and of families (by genus).”

- The method for MS/MS molecular networking is described but the resulting network is not curated in the main text. Thus, I doubt if the method needs to be included here.

We are indeed not depicting the molecular network in this paper for several reasons. First it is very massive (>110000 nodes) and thus static depictions are hardly usable. The second reason is linked to the previous one, indeed it is because of the size of such a network that we employed TMAP based visualization of the spectral space and these figures are used in the paper. We however believe that the interactive exploration of molecular networks is very efficient to navigate through chemical families. We thus added a “ready to explore” cytoscape file corresponding to a fully annotated molecular network and the following line in the main text : “A Cytoscape file corresponding to the full molecular network mapped with a color layout corresponding to NPClassifier chemical classification, the experimental and theoretical spectral matches as well as a feature table grouped at the family level is available through the following MassIVE repository link.”

- It is interesting that flavonoids showed relatively low coverage because it is one of the most commonly distributed classes of phytochemicals. Could the authors discuss on this?

Here we invite the reviewer to have a closer look at the following interactive barplot https://mandelbrot-project.github.io/pf_1600_datanote/barchart_superclass.html (also linked in the main text). It can be observed that flavonoids are indeed among the large superclass in terms of diversity. The bar plot indicates that the coverage at this specific class is slightly over 41%, larger superclass such as triterpenoids (also very widespread) indicate a coverage of 38 % so overall quite comparable and not particularly low for this specific class of compounds.

- In page 13, some subfigures are indexed wrong. 1B and 1C should be revised to 3B and 3C, respectively.

Thank you for pointing out this error. It has been corrected in the main text.

- Some citations, especially patents, need more bibliographic information.

Thanks for spotting these errors. The bibliography should now be up to date.

Reviewer #2: Allard et al. present a public dataset of plant extracts and their initial exploration using computational tools to mine this dataset. The authors aim to frame this as just the beginning in extracting all the discovery potential from this dataset.

Main Comments

Please include a citation for the MEMO tool.

The citation for the MEMO tool is appearing on page 9. See “Applied to the current plant extract collection, MEMO allows efficient reduction of the observable batch-effect and clustering samples according to their content [18].”

I would recommend a more detailed description of the usage of the TMAP visualization. For example, what exactly is the score of peaks and losses between MS/MS spectra. Was it the aligned cosine used in the molecular networking or something more akin to a shared peak count?

Powered by Editorial Manager® and ProduXion Manager® from Aries Systems Corporation

	We thank the reviewer for this question and have detailed the process of the TMAP construction both for the spectral and structural based TMAPs. See main text e.g. “In addition to the MN, spectra were organized using TMAP visualization [20]. Briefly, a minimum spanning tree is built from a network of spectral similarity. For this, spectra were first translated to documents (two decimals were used, i.e. a peak at 100.3897 would be translated as “peak@100.39”) using matchms and spec2vec packages (Huber et al. 2020; Huber et al. 2021), with calculation of neutral losses (of up to 400 m/z) to the precursor. The spectral documents were then hashed using the MinHash scheme and indexed in an LSH forest that was used to generate the TMAP visualization based on the presence/absence of peaks and losses in spectra [21] and [24].”<br><br>Additionally, it seems implied that the TMAP is based upon the clustered MS/MS spectra produced by the MZMine tool, but should be made more explicit.<br><br>Thank you. This was specified in the main text and a link was added. See “Here the spectral list corresponding to the aligned feature table produced by MzMine was employed.” Please note that the links to the input file are equally present in the TMAP generating scripts.<br><br>The text for the section "Visualization of the metabolite annotations" is rather confusing and I actually cannot parse out the specific meaning here. I think the message should be related to Figure 3, where a TMAP is created utilizing the structure similarity of the putative annotations. However, the mixing of references to Figure 1 is rather confusing and I would recommend sticking with Figure 3's visualizations in carrying the story forward.<br><br>Indeed, this was also mentioned by the other reviewer. We corrected the reference to the Figure and the text should now read better.<br><br>Minor Comments<br><br>Correction in abstract, should be: "Researchers interested in the exploitation of large and chemodiverse extracts collections should use elaborate strategies to efficiently tackle the chemical complexity and access these structures. "<br><br>Here we wanted to use “elaborate” as a verb. We changed the sentence to “Researchers interested in the exploration of large and chemodiverse extracts collections should thus establish strategies aiming to efficiently tackle such chemical complexity and access these structures.”<br><br>Overall, I believe the authors limit their claims on this manuscript which is much in line with the results presented. It is not meant to be a final analysis but just the start. I do think some of the results language and presentation needs to be tightened up, as some parts as noted above are hard to comprehend and as a reader am a little confused as the message. As the authors are already limiting the scope to presenting the data, metadata (which is honestly very complete), and preliminary analysis, I think it would be good to sharpen the conclusions of the initial analysis since the merits of this paper does not rest upon an extensive results section.<br><br>We thank the reviewer for this comment. We have added a Data availability and reusability section in the main text and before the conclusion. This section showcases some reuse scenarios for this dataset and should hopefully clarify the message of the paper : “Here is a large metabolomics dataset which was fully annotated by the authors. Scripts, methods and results are made available to the community. Now it is open to further reanalysis. We are very much looking forward for such reutilisation by others !”
**Additional Information:**	
**Question**	**Response**
Are you submitting this manuscript to a special series or article collection?	No

Powered by Editorial Manager® and ProduXion Manager® from Aries Systems Corporation

**Experimental design and statistics**<br><br>Full details of the experimental design and statistical methods used should be given in the Methods section, as detailed in our Minimum Standards Reporting Checklist. Information essential to interpreting the data presented should be made available in the figure legends.<br><br>Have you included all the information requested in your manuscript?	No
If not, please give reasons for any omissions below.<br><br>as follow-up to "**Experimental design and statistics**<br><br>Full details of the experimental design and statistical methods used should be given in the Methods section, as detailed in our Minimum Standards Reporting Checklist. Information essential to interpreting the data presented should be made available in the figure legends.<br><br>Have you included all the information requested in your manuscript?<br><br>"	Not applicable. Not statistical analysis in this datanote.
**Resources**<br><br>A description of all resources used, including antibodies, cell lines, animals and software tools, with enough information to allow them to be uniquely identified, should be included in the Methods section. Authors are strongly encouraged to cite Research Resource Identifiers (RRIDs) for antibodies, model organisms and tools, where possible.<br><br>Have you included the information	Yes

Powered by Editorial Manager® and ProduXion Manager® from Aries Systems Corporation

requested as detailed in our Minimum Standards Reporting Checklist?	
**Availability of data and materials**<br><br>All datasets and code on which the conclusions of the paper rely must be either included in your submission or deposited in publicly available repositories (where available and ethically appropriate), referencing such data using a unique identifier in the references and in the “Availability of Data and Materials” section of your manuscript.<br><br>Have you have met the above requirement as detailed in our Minimum Standards Reporting Checklist?	Yes

Manuscript Click here to access/download;Manuscript;PF_datanote.pdf

Open and re-usable annotated mass spectrometry dataset of a chemodiverse collection of 1,600 plant extracts.

Pierre-Marie Allard[1,2,3], pierre-marie.allard@unifr.ch, 0000-0003-3389-2191
Arnaud Gaudry[1,2], arnaud.gaudry@unige.ch, 0000-0002-3648-7362
Luis-Manuel Quirós-Guerrero[1,2], luis.guerrero@unige.ch, 0000-0002-1630-8697
Adriano Rutz[1,2], adriano.rutz@unige.ch, 0000-0003-0443-9902
Miwa Dounoue-Kubo[4], miwa-k@ph.bunri-u.ac.jp, 0000-0002-7271-3128
Tom W. N. Walker[5], thomas.walker@unine.ch, 0000-0001-8095-6026
Emmanuel Defossez[3,5], emmanuel.defossez@unifr.ch, 0000-0002-3279-9190
Christophe Long[6], christophe.long@pierre-fabre.com, 0000-0002-7677-7700
Antonio Grondin[7], antonio.grondin@pierre-fabre.com, 0000-0002-2952-6271
Bruno David[7], brunoxdavid@gmail.com, 0000-0002-6222-9228
Jean-Luc Wolfender[1,2], jean-luc.wolfender@unige.ch, 0000-0002-0125-952X

[1]Institute of Pharmaceutical Sciences of Western Switzerland, University of Geneva, 1211 Geneva 4, Switzerland
[2] School of Pharmaceutical Sciences, University of Geneva, 1211 Geneva 4, Switzerland
[3] Department of Biology, University of Fribourg, 1700 Fribourg, Switzerland
[4] Faculty of Pharmaceutical Sciences, Tokushima Bunri University, 180 Yamashiro-cho, Tokushima, 770-8514, Japan.
[5] Institute of Biology, University of Neuchâtel, Neuchâtel, Switzerland
[6] Direction Scientifique Naturactive, Pierre Fabre Medicament, 17 Avenue Jean Moulin, 81100 Castres, France.
[7] Green Mission Pierre Fabre, Institut de Recherche Pierre Fabre, 3 Avenue Hubert Curien, BP 13562, 31562 Toulouse, France.

Abstract

As privileged structures, natural products often display potent biological activities. However, the discovery of novel bioactive scaffolds is often hampered by the chemical complexity of the biological matrices they are found in. Large natural extracts collections are thus extremely valuable for their chemical novelty potential but also complicated to exploit in the frame of drug-discovery projects. In the end, it is the pure chemical substances that are desired for structural determination purposes and bioactivity evaluation. Researchers interested in the exploration of large and chemodiverse extracts collections should thus establish strategies aiming to efficiently tackle such chemical complexity and access these structures. Establishing carefully crafted digital layers documenting the spectral and chemical complexity as well as bioactivity results of natural products extracts collections can help to prioritize time-consuming but mandatory isolation efforts. In this note, we report the results of our initial exploration of a collection of 1,600 plant extracts in the frame of a drug discovery effort. After describing the taxonomic coverage of this collection, we present the results of its liquid chromatography high-resolution mass spectrometric profiling and the exploitation of these profiles using computational solutions. The resulting annotated mass spectral dataset and associated chemical and taxonomic metadata are made available to the community and data reuse cases are proposed. We are currently continuing our exploration of this plant extracts collection for drug-discovery purposes (notably looking for novel anti-trypanosomatids, anti-infective and prometabolic compounds) and eco-metabolomics insights. We believe that such a dataset can be exploited and reused by researchers interested in computational natural products exploration.

Keywords

Plant extracts collection, metabolomics, drug discovery, LC-MS, natural products, mass spectrometry, chemodiversity, open-science, biodiversity digitization

Data Description

In the frame of a partnership between academia and industry, a collaboration was established in 2017 between the Laboratory of Phytochemistry and Bioactive Natural Products at the University of Geneva (LPBNP), Switzerland, and the Pierre Fabre Laboratories (PFL) in Toulouse, France. This collaboration had the objective to explore and exploit the chemodiversity of a large collection of plant extracts (furnished by the industrial partner) using state-of-the-art mass spectrometric profiling methods and associated data mining solutions (performed and developed by the academic partner). The main goal of this research project is to establish a precise and exploitable description of the wide chemical diversity displayed in the plant extracts collection in order to orient further isolation efforts aiming to discover, in a first move, novel anti-trypanosomatids scaffolds and deepen chemotaxonomic knowledge of such a large set.

Context

Pierre Fabre Laboratories were founded in 1962 by Pierre Fabre, a French pharmacist from Castres (Tarn, France). They have specialized since the very beginning in the exploration and valorization of plants as medicines, health, and beauty products. Innovation based on the vegetal world in PFL encompasses chemotherapy treatments with vinca alkaloids extracted from the leaves of *Catharanthus roseus* (Q161093) [1], dermo-cosmetic products such as hair dyes from *Lawsonia inermis* (Q182448) extracts [2], or celastrol (Q5057534)-enriched extracts for psoriatic skin obtained from in vitro plant cell culture of *Tripterygium wilfordii* (Q1424919) [3]. In 1998, Pierre Fabre decided to launch a High Throughput Screening (HTS) program based on plant extracts with the objective to find novel anti-cancer drugs. The first samples of plant parts were collected in 1998 and when the HTS program ended in December 2015, the collection contained just over 17,000 unique samples. Multiple scientific articles originating from the exploration of this unique collection have been published over those years [4–7]. Hereafter is a selection of natural products-based scaffolds that entered antitumor medicinal chemistry programs: dimeric derivatives of artemisinin (Q426921) [8]; flavagline derivatives (Q3073444) [9]; griseofulvin (Q416096) [10]; narciclasine (Q18379239) and pancratistatin (Q7130395) [11]; neoboutomellerone [12]; triptolide (Q906351) [13].

The PFL collection, which is among the largest collection of plant samples worldwide with over 17,000 unique samples, was registered on April 1, 2020 at the European Commission under the accession number 03-FR-2020. This official registration recognizes the legality of the access and management process. It means that the collection meets the criteria set out in the EU ABS Regulation which implements at

the European level the requirements of the Nagoya Protocol regarding access to genetic resources and the fair and equitable sharing of benefits arising from their utilization [14]. To date three european collections are recognized (https://ec.europa.eu/environment/nature/biodiversity/international/abs/pdf/Register%20of%20Collections.pdf).

In 2015 the Nature Open Library program was launched in order to share the unique PFL expertise with industrial or academic partners and to foster the research, development and industrialization of plant assets. In this context, PFL provided access to their private plants' collection, including some rare species. (https://www.pierre-fabre.com/en/press_release/pierre-fabre-laboratories-unveils-open-nature-library-a-unique-open-innovation). Upon restructuration, this program was discontinued as of 2018. Some academic collaborations established at this time were kept active. The partnership established between LPBNP and PFL in 2017 had for principal objective the chemical characterization of the full PFL plant extracts collection. In order to evaluate the feasibility of such an ambitious program, a pilot project was defined. It focussed on a selection of 1,600 samples (corresponding to approximately 10% of the extracts prepared in the full PFL collection). The results of this pilot study, namely the generated data and outcomes are shared in this data note.

Note : from now on, in this manuscript, the full collection (17,000 extracts) is referred to as the "*PFL collection*". The selected set is simply referred to as "*the 1,600 extracts collection*".

Sample collection

All samples from the PFL collection were collected with respect to the different regulations around genetic resources at the time of collection. After drying and grounding, they were stored at room temperature, in the dark, in high-density polyethylene (HDPE) bar-coded 0.5L or 2L-pots. The storage room is access secured and protected by an automatic fire protection system with inert gas (50% nitrogen, 50% argon). Precise localization of the geographical sampling sites, unique IDs, barcodes and quantities, are stored in the PFL internal data management system. Furthermore, to perform later sample inspections, for example in case of dubious botanical identifications, all dry plant parts were also sampled and stored in their intact form. Several constraints were followed while building this collection. First, to have the best chances of finding new chemical entities, a large chemical diversity was desirable. Taking the assumption that taxonomical position and chemical production were related, samples were collected in order to maximize diversity within superior plant taxa (classes, orders, family and genera, see Figure 1). Second, only plants providing reasonable biomass quantities (i.e. > 50 g dry ground samples) were

collected, hence allowing isolation of potentially active compounds in sufficient quantities to be characterized and to perform preliminary bioassays. Third, for preserving samples on the long term but also for allowing only stable compounds to be left in the samples, they were dried for three days at 55°C. This point foresaw issues of potential HTS hits due to unstable compounds which would be difficult to isolate and manipulate.

The phylogenetic coverage of the 1,600 extracts collection is depicted in Figure 1. Within the Streptophyta ([Q133527]()) phylum the collection represented ca. 64 % of the known orders and ca. 30 % of all known botanical families. The numbers rapidly decreased to ca. 4 % of the known genus and less than 0.5 % of all known species. See horizontal bar plot on Figure 1. The botanical families of the collection are relatively well distributed across the global phylogeny, despite some orders (e.g. Sapindales ([Q26316]()), Rosales ([Q21895]())) being better covered than others (e.g. Crossosomatales ([Q21860]()), Cyatheales ([Q623232]())). Interactive bar plots are available for the inspection of the coverage of [orders]() (by families) and of [families]() (by genus). The scripts for taxonomical names resolving and figure generation are available at https://github.com/mandelbrot-project/pf_1600_datanote/blob/main/src/phylogenetic_coverage.R

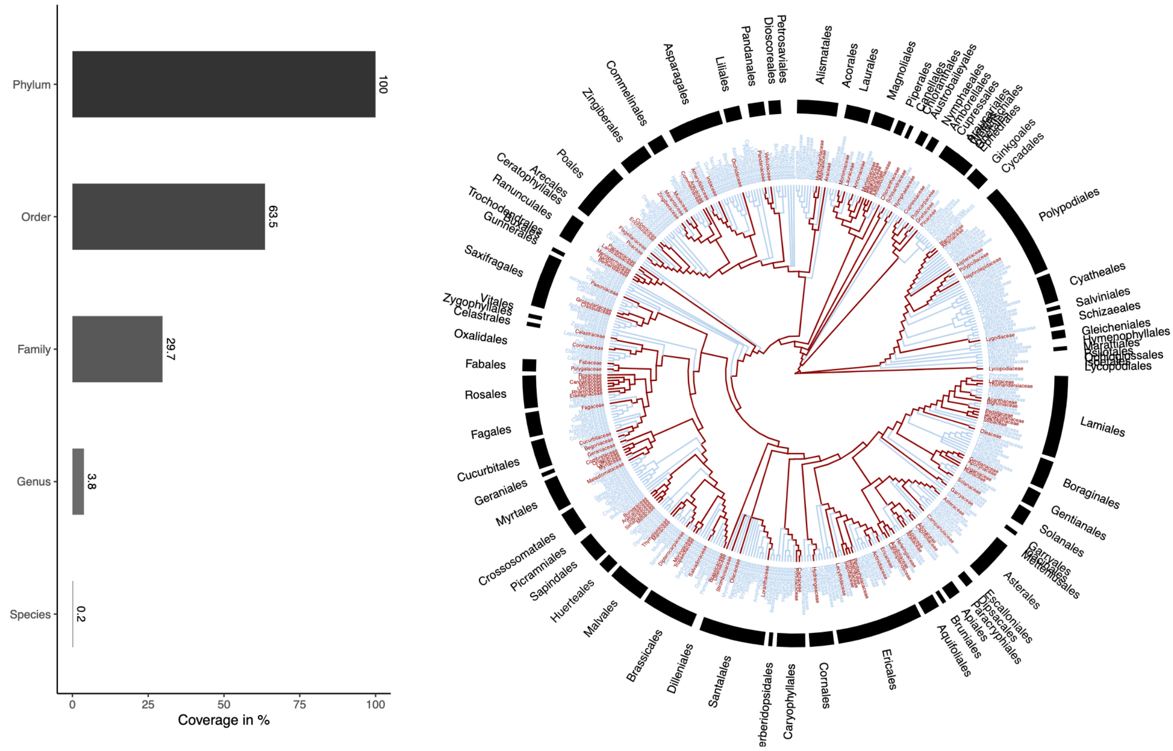

Figure 1: Taxonomical coverage of the profiled collection (1,600). On the left, the bar plot represents the overall coverage at main taxa level and up to the phylum Streptophyta. On the right, the taxonomical coverage is represented using a taxonomical tree of all families within the Streptophyta phylum. The families present within the current collection are highlighted in red. The Open Tree of Life (ott3.3) was used for taxonomy resolving.

Extraction and sample preparation

Since the PFL collection was designed to provide extracts for HTS experiments, only medium polarity compounds were targeted. Using ethyl acetate (EtOAc) extracts purified over silica gel allowed to reduce the amount of known classes of pan-assay interference compounds (PAINS) such as condensed tannins [15].

Starting from the dried plant material, the extracts were prepared as follows. A sample of 8 g (leaves, whole plant or aerial parts) or 10 g (subterranean parts, roots, bark) was extracted over a period of 8-10 h with 80 mL or 100 mL of EtOAc respectively. The extracts were filtered through paper and dried under vacuum in a rotary evaporator to a reduced volume (ca. 5 mL). The residue was transferred to a pre-weighted recipient and dried under vacuum until measurement of constant weight. The extracts were dissolved in EtOAc at a concentration of 30 mg/mL. An aliquot of 1 mL was mixed with 200 mg of silica gel (Merk 60, 40-63 μm) and dried under vacuum. The resulting powder mixture was transferred to a SPE cartridge (6 mL, 1 g SiO2). The cartridge was eluted with 10 mL of dichloromethane to remove apolar compounds, then the cartridge was eluted with 8 mL of a mixture dichloromethane:methanol (85:15), and subsequently washed with 2 mL of the same mixture to fully recover the compounds of interest. The filtrate (ca 10 mL final volume) was dried under vacuum. Extracts were then dissolved in DMSO to a concentration of 5 mg/mL and transferred to 1 mL 96 deep-well plates for further analysis. For LC-MS analysis (see below), the extracts were dissolved in DMSO to reach a concentration of 2.5 mg/mL. The plates originally contained 80 extracts per plate, the columns 1 and 12 being empty. These columns were filled, respectively, by a QC (quality control) sample and a DMSO blank. The QC samples were constituted by a mixture of *Cinchona pubescens* Vahl (Q164574), *Panax ginseng* C.A.Mey. (Q182881), *Ginkgo biloba* L. (Q43284), *Arnica montana* L. (Q207848) and *Salvia officinalis* L. (Q1111359) methanolic extracts dissolved in a 1:1:1:1:1 ratio at 5 mg/mL in DMSO. These blanks and QC samples were injected every ten samples.

LC-MS/MS analyses

Chromatographic separation was performed on a Waters Acquity UPLC system interfaced to a Q-Exactive Focus mass spectrometer (Thermo Scientific, Bremen, Germany), using a heated electrospray ionization (HESI-II) source. Thermo Scientific Xcalibur 3.1 software was used for instrument control. The LC conditions were as

follows: column, Waters BEH C18 50 × 2.1 mm, 1.7 μm; mobile phase, (A) water with 0.1% formic acid; (B) acetonitrile with 0.1% formic acid; flow rate, 600 $\mu L \cdot min^{-1}$; injection volume, 2 μL; gradient, linear gradient of 5−100% B over 7 min and isocratic at 100% B for 1 min. The optimized HESI-II parameters were as follows: source voltage, 3.5 kV (pos); sheath gas flow rate (N2), 55 units; auxiliary gas flow rate, 15 units; spare gas flow rate, 3.0; capillary temperature, 350.00°C, S-Lens RF Level, 45. The mass analyzer was calibrated using a mixture of caffeine, methionine–arginine–phenylalanine–alanine–acetate (MRFA), sodium dodecyl sulfate, sodium taurocholate, and Ultramark 1621 in an acetonitrile/methanol/water solution containing 1% formic acid by direct injection. The data-dependent MS/MS events were performed on the three most intense ions detected in full scan MS (Top3 experiment). The MS/MS isolation window width was 1 Da, and the stepped normalized collision energy (NCE) was set to 15, 30 and 45 units. In data-dependent MS/MS experiments, full scans were acquired at a resolution of 35,000 FWHM (at *m/z* 200) and MS/MS scans at 17,500 FWHM both with an automatically determined maximum injection time. After being acquired in a MS/MS scan, parent ions were placed in a dynamic exclusion list for 2.0 s.

Data-processing and Molecular Networking

The MS data were converted from .RAW (Thermo) standard data format to .mzXML format using the MSConvert software, part of the ProteoWizard package [16]. The converted files were treated using the MZMine software suite v. 2.53 [17]. The parameters were adjusted as follows: the centroid mass detector was used for mass detection with the noise level set to 1.0E4 for MS level set to 1, and to 0 for MS level set to 2. The ADAP chromatogram builder was used and set to a minimum group size of scans of 5, minimum group intensity threshold of 1.0E4, minimum highest intensity of 5.0E5 and *m/z* tolerance of 12 ppm [18]. For chromatogram deconvolution, the algorithm used was the wavelets (ADAP). The intensity window S/N was used as S/N estimator with a signal to noise ratio set at 10, a minimum feature height at 5.0E5, a coefficient area threshold at 130, a peak duration ranges from 0.0 to 0.5 min and the RT wavelet range from 0.01 to 0.03 min. Isotopes were detected using the isotopes peaks grouper with a *m/z* tolerance of 12 ppm, a RT tolerance of 0.01 min (absolute), the maximum charge set at 2 and the representative isotope used was the most intense. Each feature list was filtered before alignment to keep only features with an associated MS2 scan and a RT between 0.5 and 8.0 min using the feature filtering. Peak alignment was performed using the join aligner method (*m/z* tolerance at 40 ppm), absolute RT tolerance 0.2 min, weight for *m/z* at 2 and weight for RT at 1 and a weighted dot-product cosine similarity of 0.3. The aligned feature list (119,182 features) was exported using the export to GNPS module. Features occurring in QC

samples only or in blanks were removed before molecular networking, resulting in a final feature list of 117,005 features. The MZMine parameters used for the data treatment are available at https://massive.ucsd.edu/ProteoSAFe/DownloadResultFile?file=f.MSV000087728/updates/2022-05-02_pmallard_e88304cd/other/210302_VGF_pos_parameters.xml

The initial study of this aligned peak list with classical unsupervised statistics (e.g. PCA, PCoA) indicated a strong batch effect which could be tracked down to a specific date and attributed to a change of column during the course of the mass spectrometry analysis of the full collection. Such batch effects are almost inevitable when samples are profiled on long periods (here spanning over several months) and are particularly complicated to mitigate especially when dealing with chemodiverse datasets where poor overlap among the samples is expected. This incentivized us to develop a novel computational mass spectrometry solution for MS2 BasEd SaMple VectOrization (MEMO). MEMO allows to organize samples in large and chemodiverse collections in a retention-time agonist fashion thus strongly mitigating batch effect and allowing the comparison of samples acquired over heterogeneous chromatographic conditions. Applied to the current plant extract collection, MEMO allows efficient reduction of the observable batch-effect and clustering samples according to their content [19]. See the following link for a view of the batch effect on the classically aligned dataset and its mitigation using MEMO fingerprints (https://mandelbrot-project.github.io/memo_publication_examples/plant_extract_dataset/pcoa_vgf_color_before_after.html)

To analyze the spectral diversity of the profile collection, a molecular network (MN) was created using the online workflow (https://ccms-ucsd.github.io/GNPSDocumentation/) on the GNPS website (http://gnps.ucsd.edu) using the .mgf spectra file generated at the previous step [20]. The precursor ion mass tolerance was set to 0.02 Da and a MS/MS fragment ion tolerance of 0.02 Da. A network was then created where edges were filtered to have a cosine score above 0.7 and more than 6 matched peaks. Further, edges between two nodes were kept in the network if and only if each of the nodes appeared in each other's respective top 10 most similar nodes. Finally, the maximum size of a spectral family was set to 100, and the lowest scoring edges were removed from molecular families until the molecular family size was below this threshold. The spectra in the network were then searched against GNPS spectral libraries. All matches kept between network spectra and library spectra were required to have a score above 0.7 and at least 6 matched peaks. The resulting molecular network is available at https://proteomics2.ucsd.edu/ProteoSAFe/status.jsp?task=3197f70bed224f9ba6f59f62906839e9. A Cytoscape file corresponding to the full molecular network mapped with

a color layout corresponding to NPClassifier chemical classification, the experimental and theoretical spectral matches as well as a feature table grouped at the family level is available through the following MassIVE repository link.

Via the GNPS Explorer interface it is possible to efficiently navigate through the uploaded spectral file and their associated metadata. See this link https://gnps-explorer.ucsd.edu/MSV000087728?dataset_accession=MSV000087728&metadata_source=MASSIVE&metadata_option=f.MSV000087728%2Fupdates%2F2021-11-15_pmallard_334e9199%2Fmetadata%2Fgnps_metadata.tsv. For example, the name of a plant of interest can be typed under the NCBI Taxonomy header resulting in the direct filtering of the dataset for this specific plant. Individual spectral files can then be selected (and eventually compared if multiple are selected) and viewed using the GNPS dashboard. See for example this interactive view of the total ion chromatogram of *Desmodium heterophyllum* Hook. & Arn (Q10770714) aerial parts.

In addition to the MN, spectra (from the same .mgf file used for MN) were organized using TMAP visualization [21]. In this case the TMAP corresponds to a minimum spanning tree built from a dense network of spectral similarity. For the establishment of this visualization, spectra were first translated to documents (two decimals were used, i.e. a peak at 100.3897 would be translated as "peak@100.39") using matchms and spec2vec packages [22,23], with calculation of neutral losses (up to 400 *m/z*) to the precursor. The spectral documents, i.e. a list of peaks and losses without intensity information, were then hashed using the MinHash scheme and indexed in an LSH forest that was used to generate the TMAP visualization based on the presence/absence of peaks and losses in spectra [21,24]. The generated TMAP is presented in Figure 2, an interactive visualization is available at https://mandelbrot-project.github.io/pf_1600_datanote/220408_VGF_samples_spectral_tmap_pos.html, and the code used for the TMAP generation is available at https://github.com/mandelbrot-project/pf_1600_datanote/blob/main/src/spectral_tmap.py. Such visualization allows to highlight taxa-specific spectral subspaces. In addition, structural annotation (see next section for details concerning the metabolite annotation process) were overlaid to their corresponding spectra. Here structural annotations were limited to compounds reported in the Eukaryote domain. For example the spectra region 1 in Figure 2 B. and 2 D. is mainly specific to samples belonging to the Meliaceae (Q158979) family and most of these spectra are annotated as limonoids derivatives (Q669514). Another region, called region 2 in Figure 2, contains mainly spectra specific to the Annonaceae (Q220025) family and corresponds to acetogenins derivatives (Q3604300). Finally, the spectral region 3 is specific to the Apocynaceae family (Q173756) and is mainly annotated as tryptophan alkaloids derivatives. On the other hand, botanical families such as the Fabaceae (Q44448) appear to occupy a

much wider spectral space. These examples showcase the importance of the taxonomic coverage of an extract collection to maximize the chemical diversity. Indeed, if some chemical classes, such as the flavonoid (Q3561192) derivatives, appear to be widespread, some others are known to be extremely specific to some taxa, such as the acetogenins to the Annonaceae [25]. Of course, the interpretation of this spectral TMAP is highly dependent on the structural annotations' quality (see next section "Metabolite annotation") and as such should be taken with caution. Because the annotation process favored structures reported in closer taxa, it should also be noted that the comparison of annotation occurrences and taxonomy may be impacted by an annotation set biased towards denser taxa-specific ensembles of structures.

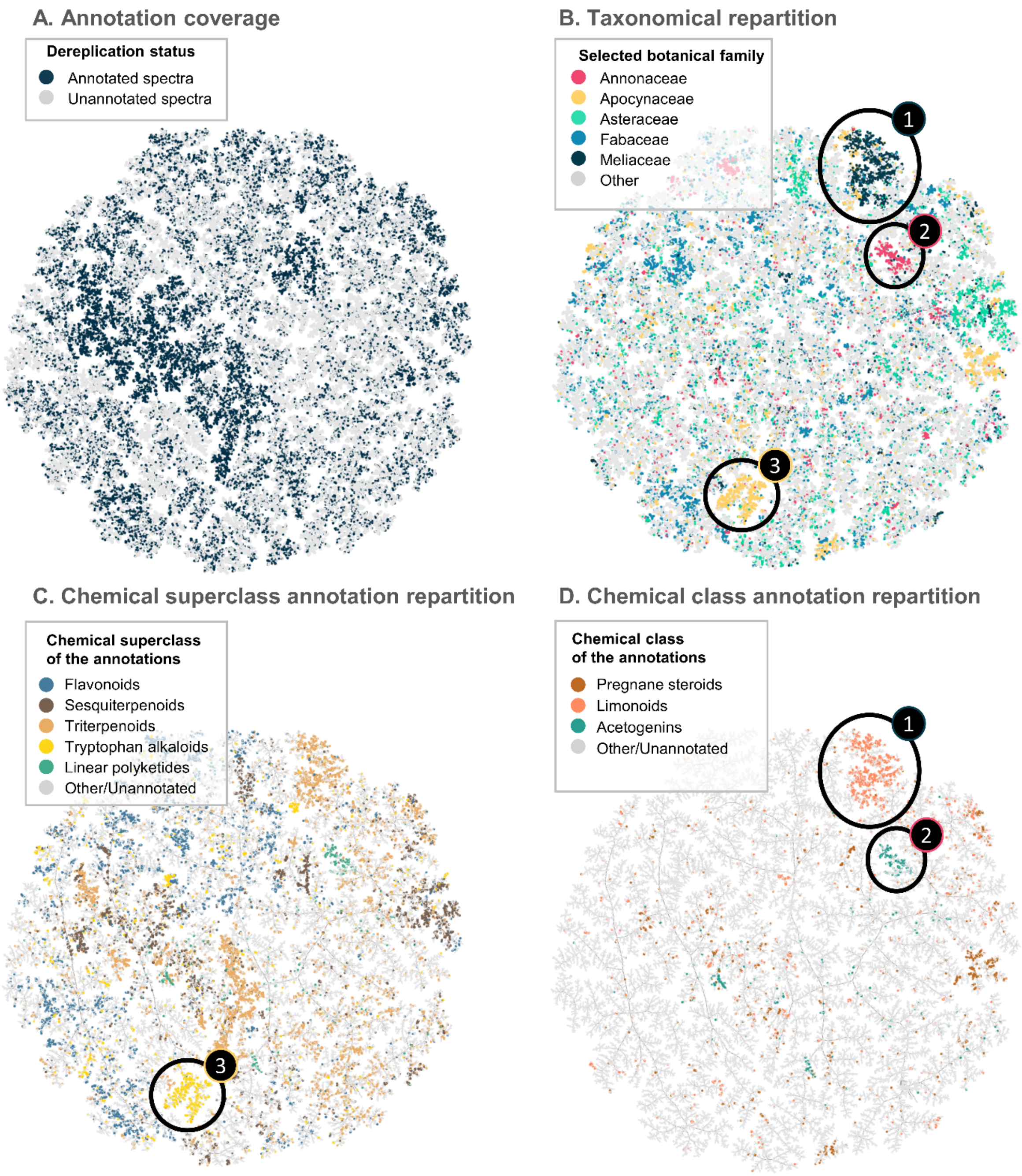

Figure 2: Spectral diversity of the profiled plant collection (1,600 extracts). The TMAP approach is employed to display the >100,000 of spectra resulting from the alignment of the 1,600 untargeted MS/MS experiments. In this TMAP each dot represents a feature's spectrum and they are linked together according to their similarity. In **A**, blue dots (36% of the total amount of spectra) correspond to annotated spectra while gray dots (64%) correspond to unannotated spectra. In **B**, dots are colored according to the botanical family of the sample where the highest MS1 peak area for the corresponding feature was recorded. In **C** and **D**, dots are colored according to the NPClassifier superclass and class, respectively, of their annotation (for annotated dots). In **B**, it is possible to spot spectral regions (1, 2 and 3) specific to given botanical families. The region 1 is specific to the Meliaceae family and these spectra are mainly annotated as limonoids derivatives, the region 2 is specific to the Annonaceae family and spectra are mainly annotated as acetogenins derivatives and the region 3 is specific to the Apocynaceae family and spectra are annotated as tryptophan alkaloids derivatives. Note that the structural annotation results are reweighed according to the taxonomical proximity of the biological source of the candidate structure and the biological source of the annotated spectra. A bias toward taxa-specific structures can thus be observed. This interactive structural TMAP can be browsed at https://mandelbrot-project.github.io/pf_1600_datanote/220408_VGF_samples_spectral_tmap_pos.html

Metabolite annotation

Experimental spectral libraries search

The full spectral dataset corresponding to the 1,600 plant extracts collection being uploaded on the MASSIVE repository https://doi.org/doi:10.25345/C59J97, a continuous identification workflow is automatically carried against GNPS experimental spectral libraries. The latest results of this continuous identification workflow can be observed at https://gnps.ucsd.edu/ProteoSAFe/result.jsp?task=b753bf1e39cb4875bdf3b786e747bc15&view=advanced_view. The latest iteration at the time of publication (2022-04-18) indicated that a total of 2665 unique compounds were spectrally matched (see https://gnps.ucsd.edu/ProteoSAFe/result.jsp?task=ee2e8e9cf8214f48ab6ee01df652a3f2&view=all_unique_compounds)

Theoretical spectral libraries search

In addition to experimental spectral libraries search we have shown that spectral matching against theoretical spectral libraries of natural products was an efficient way to cover a much wider, yet relevant, spectral space [26]. Furthermore, we showed that taking into account the taxonomical distance between the biological source of candidate structure and the biological source of the annotated extracts greatly improved the overall quality of the annotation results [27]. Thus, in addition to the spectral search performed at the molecular networking step against publicly available spectral libraries (see previous section) a taxonomically informed metabolite

annotation was performed. For this we first established a large theoretical spectral database of natural products following a previously established metabolite annotation workflow. This spectral database and associated biological sources metadata were constructed using chemical structure and information compiled during the LOTUS Initiative's first project aiming to establish an open and evolutive ressource compiling natural products biological occurrences [28]. The theoretical spectral database is publicly available at https://doi.org/10.5281/zenodo.5607264. The biological sources metadata are available at the following address https://doi.org/10.5281/zenodo.6378204. The taxonomically informed metabolite annotation was performed using the following scripts (https://github.com/mandelbrot-project/met_annot_enhancer/releases/tag/v0.1). The parameters used for the taxonomically informed metabolite annotation process and the resulting tables can be found at https://massive.ucsd.edu/ProteoSAFe/dataset_files.jsp?task=b753bf1e39cb4875bdf3b786e747bc15#%7B%22table_sort_history%22%3A%22main.collection_asc%22%2C%22main.attachment_input%22%3A%22updates%2F2022-04-28_pmallard_b0e0f70c%22%7D. PF_full_datanote_spectral_match_results_repond.tsv corresponds to the Cytoscape formatted output.

Visualization of the metabolite annotations

In order to obtain an overview of the metabolite annotation results on the profiled collection, these were compared to the ensemble of molecules described in LOTUS, See Figure 3. For this, a TMAP [21,29,30] was built to connect similar chemical structures using the MAP4 fingerprint [31], the retrieval of the nearest neighbors was achieved as described above for the spectral TMAP construction. In Figure 3, each dot represents a chemical structure and it is linked to its neighbor according to its structural proximity. In Figure 3 A., the color code indicates the repartition of chemical structures mostly found to be present at least once in plants (green color) versus structures found only in other kingdoms (orange color). In Figure 3 B. each of the annotated structures within the 1,600 plants extracts collection is displayed (strong blue) within the rest of the reported structures in LOTUS (light gray) indicating a relatively heterogeneous structural coverage of the annotations with some denser areas. Finally, the color coding in Figure 3 C. allows to distinguish chemical classes in the overall TMAP. The NPClassifier classification is employed [32]. The framed bar plot displays the most frequently annotated chemical classes in the present dataset (strong color) versus the total count for each class in LOTUS (light color). Multiple factors can explain discrepancies between the repartition of chemical classes annotated in the 1,600 extracts collection versus the overall repetition observed in LOTUS. For example cyclic peptides (green bar in the framed bar plot) are poorly

covered. This can be explained by the fact that such structures are most often found in microbial organisms (as observed when checking this region in plot A.). On the other hand, the high coverage of limonoids in the collection can be explained by the high proportion of species from the Sapindales order (see Figure 1. and this plot) and the fact that this order is known to be the main responsible of the biosynthesis of such scaffolds [33]. An interactive version of the structural TMAP presented in Figure 3 is available at https://mandelbrot-project.github.io/pf_1600_datanote/220408_annotation_vs_lotusdnp_tmap.html

A detailed coverage of annotations of the dataset versus the full LOTUS annotations is available as interactive plots (coverage at the pathway level, at the superclass level and at the class level.)

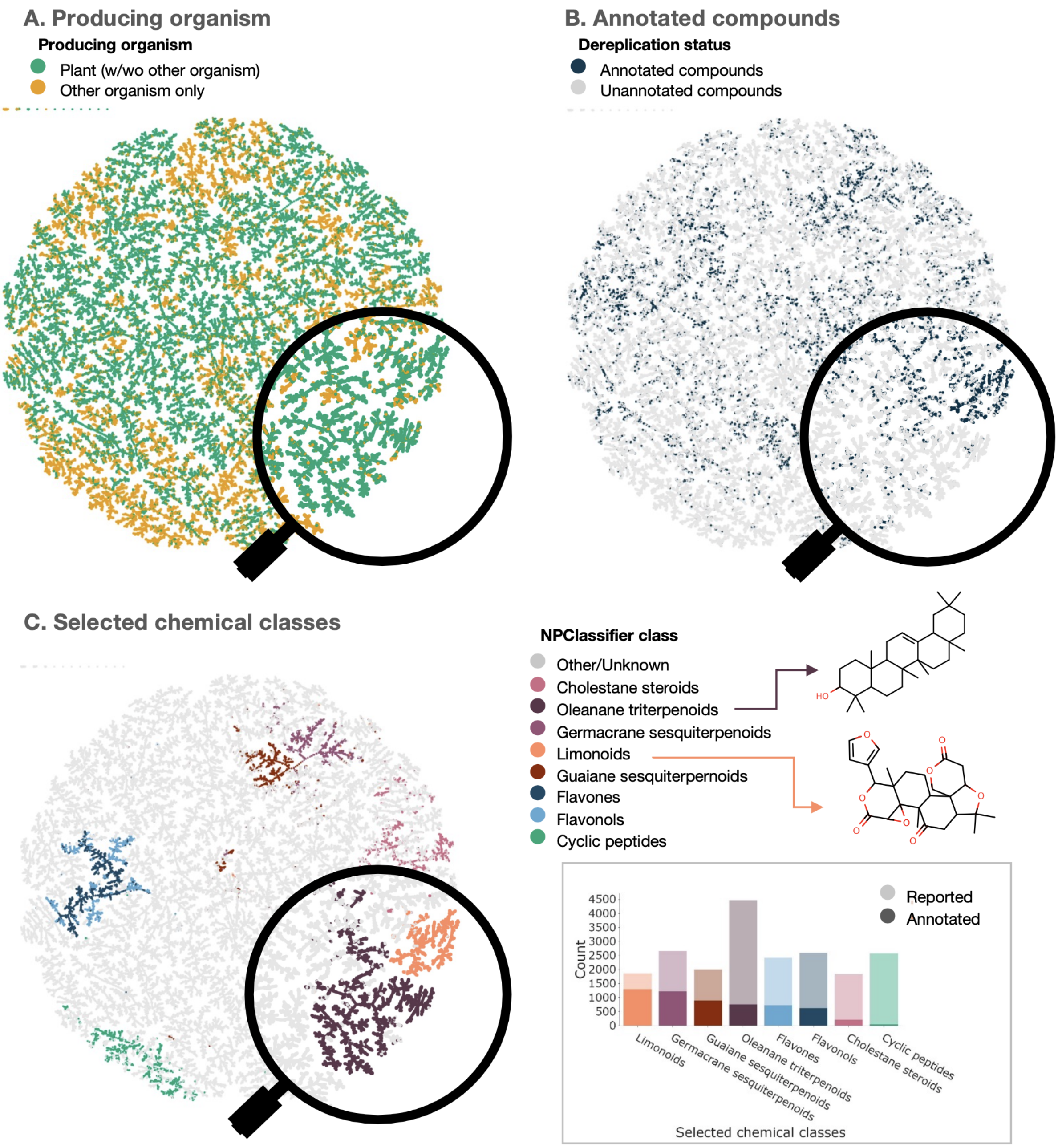

Figure 3: Chemical diversity of the profiled plant collection (1,600 extracts) and coverage against reported natural products. Visualization of reported natural products structures (LOTUS v1 and Dictionary of Natural Products v29.1) as a TMAP with plotting of the producing organism (A), the annotation's status, *i.e.* whether the 2D structure was annotated in the dataset (B) and selected NPClassifier classes (C). In the insert of C, the bar plot represents the number of compounds reported for each of the selected chemical classes with the opaque part of the bar representing the annotated compounds. The zoom on the limonoids and oleanane terpenoids clusters of the TMAP allows visualizing a well covered chemical class such as limonoids and a less covered one such as oleanane triterpenoids. A specific member of each class (limonin [Q2398745] for the limonoids and β-amyrin [Q27108621] for the oleanane triterpenoids) are represented in their planar structure form for illustration purposes. This interactive structural TMAP can be browsed at https://mandelbrot-project.github.io/pf_1600_datanote/220408_annotation_vs_lotusdnp_tmap.html

Data Availability and Reusability

Regarding data availability, all .RAW (Thermo), .mzXML and .mzML datafiles in positive and negative ionization modes along with metadata and metabolite annotation tables are available on the MassIVE repository under accession number MSV000087728, available here doi:10.25345/C59J97. Molecular networking job results are available at: https://proteomics2.ucsd.edu/ProteoSAFe/status.jsp?task=3197f70bed224f9ba6f59f62906839e9.

Regarding data reuse, multiple options exist for such a dataset. Hereafter we describe some selected use cases. For example, GNPS continuous identification can be explored and retrieved here https://gnps.ucsd.edu/ProteoSAFe/status.jsp?task=ee2e8e9cf8214f48ab6ee01df652a3f2 and can be used to compare or combine the results of experimental library matches to theoretical spectral library matches. Since the dataset is also available through the ReDu interface, multiple reanalysis possibilities can be explored. For example, through the https://redu.ucsd.edu/metadataselection interface and after selection of the PF dataset by its DatasetAccession value (MSV000087728), a filter for all files of the dataset can be established. Filters of individual species can be obtained by selecting their corresponding NCBITaxonomy terms. Later on, the full set or subset thereof, can be selected for Molecular Networking or Library Search at GNPS for example. See the complete ReDu documentation (https://mwang87.github.io/ReDU-MS2-Documentation/) for details .

The recently developed Mass Spec Query Language (MassQL) allows efficient search for specific patterns within large spectral datasets. [34] For example researchers can look for ionization patterns characteristic of dimeric monoterpene indole alkaloids (MIA) using the following MassQL query. The results of the query on the 1,600 extracts collection is available here. As an example, this query returns that a feature with a mass of 687.3570 corresponds to the searched criteria. This page displays the overlap of the MassQL query criteria and the corresponding MS1 and MS2 spectra, and also indicates that this spectra was found in sample VGF152_B02_pos.mzXML. We can then turn to the GNPS Explorer interface for the 1,600 plants dataset, filter for the sample id, and see that this sample is an extract of *Tabernaemontana coffeoides* (Q15376858) which is known to contain dimeric MIA. In the GNPS Explorer interface we can quickly filter for other species of *Tabernaemontana* (one is present, *Tabernaemontana crassa* (Q14862275)) and use the GNPS LCMS Dashboard (see plots) to observe that the incriminated feature is mostly present in *Tabernaemontana coffeoides*. A MassQL compendium groups query examples, these can be used to further explore the 1,600 extracts collection.

Conclusions

Here we present the results of state-of-the-art computational approaches used for both spectral organization and metabolite annotation of high-resolution mass spectrometry data acquired on a large collection of 1,600 chemically diverse plant extracts. All original mass spectrometry profiles and associated metadata are made available to the community for further analysis. The results of the metabolite annotation represent a putative chemical space that can be exploited and refined in subsequent drug discovery, chemotaxonomic or eco-metabolomics projects. Sharing these results with the community shows that, through effective partnership between academia and industry, the faith of historical and private collections of plant extracts can be changed and the richness of the associated chemical diversity can be made available to a wider public.

Availability of source code and requirements

The scripts used to generates the figure of this datanote are available at the following repository: https://github.com/mandelbrot-project/pf_1600_datanote

License: GNU Affero General Public License v3.0

Declarations

Competing interests

The authors declare that they have no competing interests

Funding

J-LW and P-MA are thankful to the Swiss National Science Foundation for the funding of the project (SNF N° CRSII5_189921/1).

Authors' contributions

Following the CRediT (Contributor Roles Taxonomy) roles.

Conceptualisation P-MA, BD and J-LW. Data curation P-MA, AGa, LQG, AR and MDK. Funding acquisition P-MA and J-LW. Investigation P-MA and MDK. Methodology P-MA, MDK and J-LW. Project administration P-MA, BD and J-LW. Resources CL, AGr, BD and J-LW. Software P-MA, AGa, TW, ED and AR. Supervision P-MA and J-LW. Validation P-MA, AGa and MDK. Visualization P-MA, AGa, ED and AR. Writing – original draft P-MA. Writing – review & editing P-MA, AGa, LQG, AR and AGr.

Acknowledgements

The authors are grateful to Green Mission Pierre Fabre, Pierre Fabre Research Institute, Toulouse, France for establishing and sharing this unique library of extracts.

References

1. Duflos A, Kruczynski A, Barret J-M. Novel aspects of natural and modified vinca alkaloids. *Curr Med Chem Anticancer Agents*. 2:55–702002;

2. Fiorini-Puybaret C, Joulia P. Dye composition comprising a combination of two plant extracts of lawsonia inermis. World Patent WO2020249748A1;

3. Nguyen T, Cousy A, Steward N. Method for producing celastrol and pentacyclic triterpene derivatives. World Patent WO2017194757A1;

4. Vandenberghe I, Créancier L, Vispé S, Annereau J-P, Barret J-M, Pouny I, et al.. Physalin B, a novel inhibitor of the ubiquitin-proteasome pathway, triggers NOXA-associated apoptosis. *Biochem Pharmacol*. 76:453–622008;

5. Pouny I, Long C, Batut M, Aussagues Y, Jean Valère N, Achoundong G, et al.. Quinolizidine Alkaloids from Cylicomorpha solmsii. *J Nat Prod*. 84:1198–2022021;

6. Long C, Sauleau P, David B, Lavaud C, Cassabois V, Ausseil F, et al.. Bioactive flavonoids of Tanacetum parthenium revisited. *Phytochemistry*. 64:567–92003;

7. Pouny I, Batut M, Vendier L, David B, Yi S, Sautel F, et al.. Cytisine-like alkaloids from Ormosia hosiei Hemsl. & E.H. Wilson. *Phytochemistry*. 107:97–1012014;

8. Begue J-P, Bonnet-Delpon D, Crousse B, Fournial A, Mordant C, Fahy J. Dimeric derivatives of artemisinin and application in anti-cancer therapy. World Patent WO2010012761A1;

9. Marion F, Kaloun EB, Lieby-Muller F, Perez M, Annereau J-P, Creancier L. Flavagline derivatives. World Patent WO2016001441A1;

10. Marion F, Lieby-Muller F, Grisoni S, Rahier N, Perez M, Sartori I. Griseofulvin derivatives. World Patent WO2014020101A1;

11. Marion F, Annereau J-P, Fahy J. Nitrogenated derivatives of pancratistatin. World Patent WO2010012714A1;

12. Beck J, Guminski Y, Long C, Marcourt L, Derguini F, Plisson F, et al.. Semisynthetic neoboutomellerone derivatives as ubiquitin-proteasome pathway inhibitors. *Bioorg Med Chem*. 20:819–312012;

13. Kaloun EB, Long C, Molinier N, Brel V, Cantagrel F, Massiot G. Partial synthesis of 14-deoxy-14-aminotriptolide. *Tetrahedron Lett*. 57:1895–82016;

14. : Website. http://data.europa.eu/eli/reg/2014/511/oj

15. David B, Ausseil F. High-Throughput Screening of Plant Chemodiversity. Encyclopedia of Analytical Chemistry 2014. Ed John Wiley & Sons;

16. Chambers MC, Maclean B, Burke R, Amodei D, Ruderman DL, Neumann S, et al.. A cross-platform toolkit for mass spectrometry and proteomics. *Nat Biotechnol*. 30:918–202012;

17. Pluskal T, Castillo S, Villar-Briones A, Orešič M. MZmine 2: Modular framework for processing, visualizing, and analyzing mass spectrometry-based molecular profile data. *BMC Bioinformatics*. 11:3952010;

18. Myers OD, Sumner SJ, Li S, Barnes S, Du X. One Step Forward for Reducing False Positive and False Negative Compound Identifications from Mass Spectrometry Metabolomics Data: New Algorithms for Constructing Extracted Ion Chromatograms and Detecting Chromatographic Peaks. *Anal Chem*. 89:8696–7032017;

19. Gaudry A, Huber F, Nothias L-F, Cretton S, Kaiser M, Wolfender J-L, et al.. MEMO: Mass Spectrometry-Based Sample Vectorization to Explore Chemodiverse Datasets. *Front Bioinform*. Frontiers Media SA; 2022; doi: 10.3389/fbinf.2022.842964.

20. Wang M, Carver JJ, Phelan VV, Sanchez LM, Garg N, Peng Y, et al.. Sharing and community curation of mass spectrometry data with GNPS. *Nat Biotechnol*. 34:828–372016;

21. Probst D, Reymond J-L. Visualization of very large high-dimensional data sets as minimum spanning trees. *J Cheminform*. 12:122020;

22. Huber F, Verhoeven S, Meijer C, Spreeuw H, Castilla E, Geng C, et al.. Matchms - processing and similarity evaluation of mass spectrometry data. *J Open Source Softw*. The Open Journal; 5:24112020;

23. Huber F, Ridder L, Verhoeven S, Spaaks JH, Diblen F, Rogers S, et al.. Spec2Vec: Improved mass spectral similarity scoring through learning of structural relationships. *PLoS Comput Biol*. 17:e10087242021;

24. : [No title]. ACM Digital Library. https://dl.acm.org/doi/10.1145/1060745.1060840 Accessed 2022 Sep 11.

25. Neske A, Ruiz Hidalgo J, Cabedo N, Cortes D. Acetogenins from Annonaceae family. Their potential biological applications. *Phytochemistry*. 174:1123322020;

26. Allard P-M, Péresse T, Bisson J, Gindro K, Marcourt L, Pham VC, et al.. Integration of Molecular Networking and In-Silico MS/MS Fragmentation for Natural Products Dereplication. *Anal Chem*. 88:3317–232016;

27. Rutz A, Dounoue-Kubo M, Ollivier S, Bisson J, Bagheri M, Saesong T, et al.. Taxonomically Informed Scoring Enhances Confidence in Natural Products

Annotation. *Front Plant Sci*. 10:13292019;

28. Rutz A, Sorokina M, Galgonek J, Mietchen D, Willighagen E, Gaudry A, et al.. The LOTUS initiative for open knowledge management in natural products research. *eLife*. 2022; doi: 10.7554/eLife.70780.

29. Probst D, Reymond J-L. SmilesDrawer: Parsing and Drawing SMILES-Encoded Molecular Structures Using Client-Side JavaScript. *J Chem Inf Model*. 58:1–72018;

30. Probst D, Reymond J-L. FUn: a framework for interactive visualizations of large, high-dimensional datasets on the web. *Bioinformatics*. 34:1433–52018;

31. Capecchi A, Probst D, Reymond J-L. One molecular fingerprint to rule them all: drugs, biomolecules, and the metabolome. *J Cheminform*. 12:432020;

32. Kim HW, Wang M, Leber CA, Nothias L-F, Reher R, Kang KB, et al.. NPClassifier: A Deep Neural Network-Based Structural Classification Tool for Natural Products. *J Nat Prod*. 84:2795–8072021;

33. Hodgson H, De La Peña R, Stephenson MJ, Thimmappa R, Vincent JL, Sattely ES, et al.. Identification of key enzymes responsible for protolimonoid biosynthesis in plants: Opening the door to azadirachtin production. *Proc Natl Acad Sci U S A*. 116:17096–1042019;

34. Jarmusch AK, Aron AT, Petras D, Phelan VV, Bittremieux W, Acharya DD, et al.. A Universal Language for Finding Mass Spectrometry Data Patterns. bioRxiv.

Figure 1

Click here to access/download;Figure;figure_1.pdf

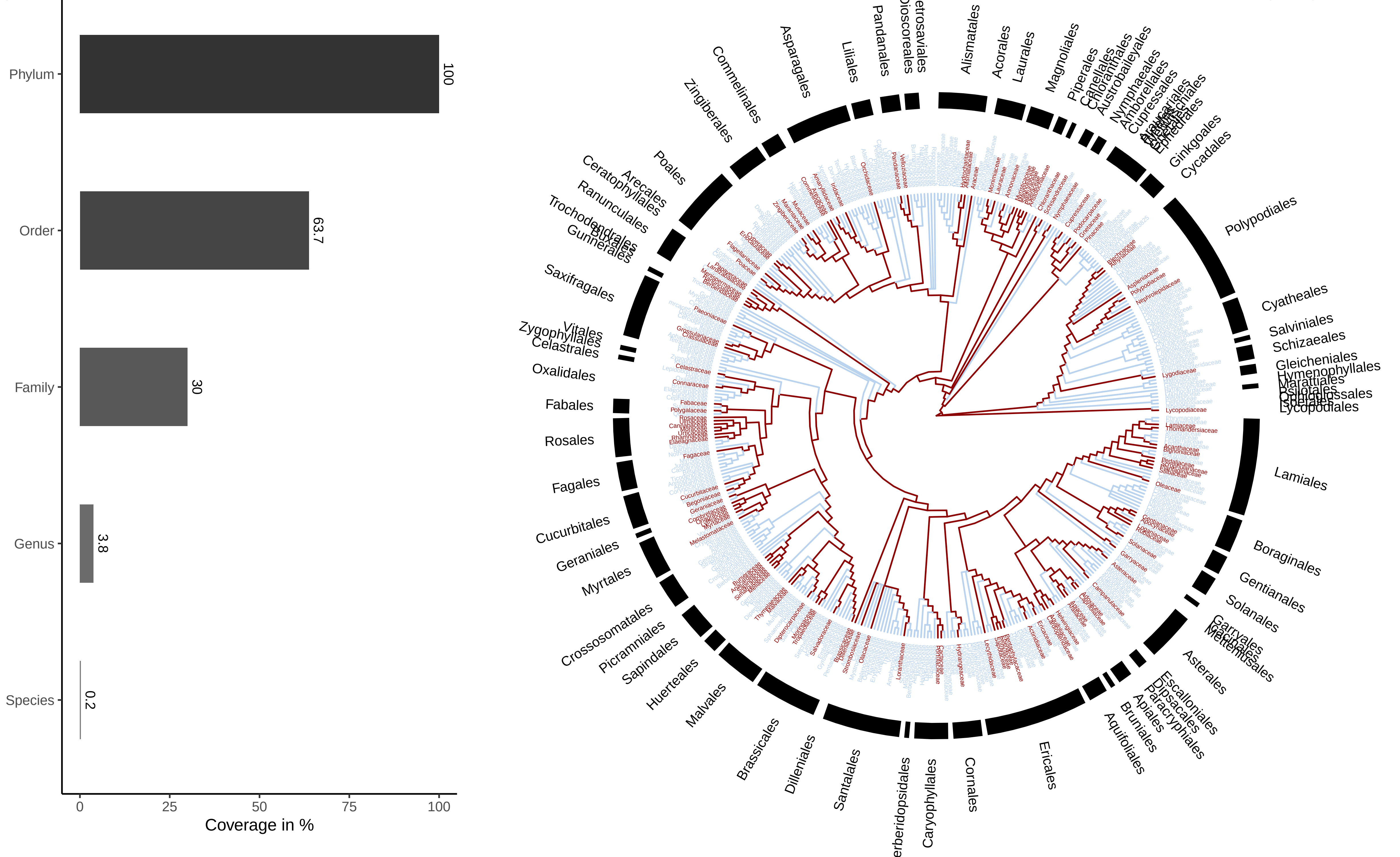

Figure 2

Click here to access/download;Figure;figure_2.pdf

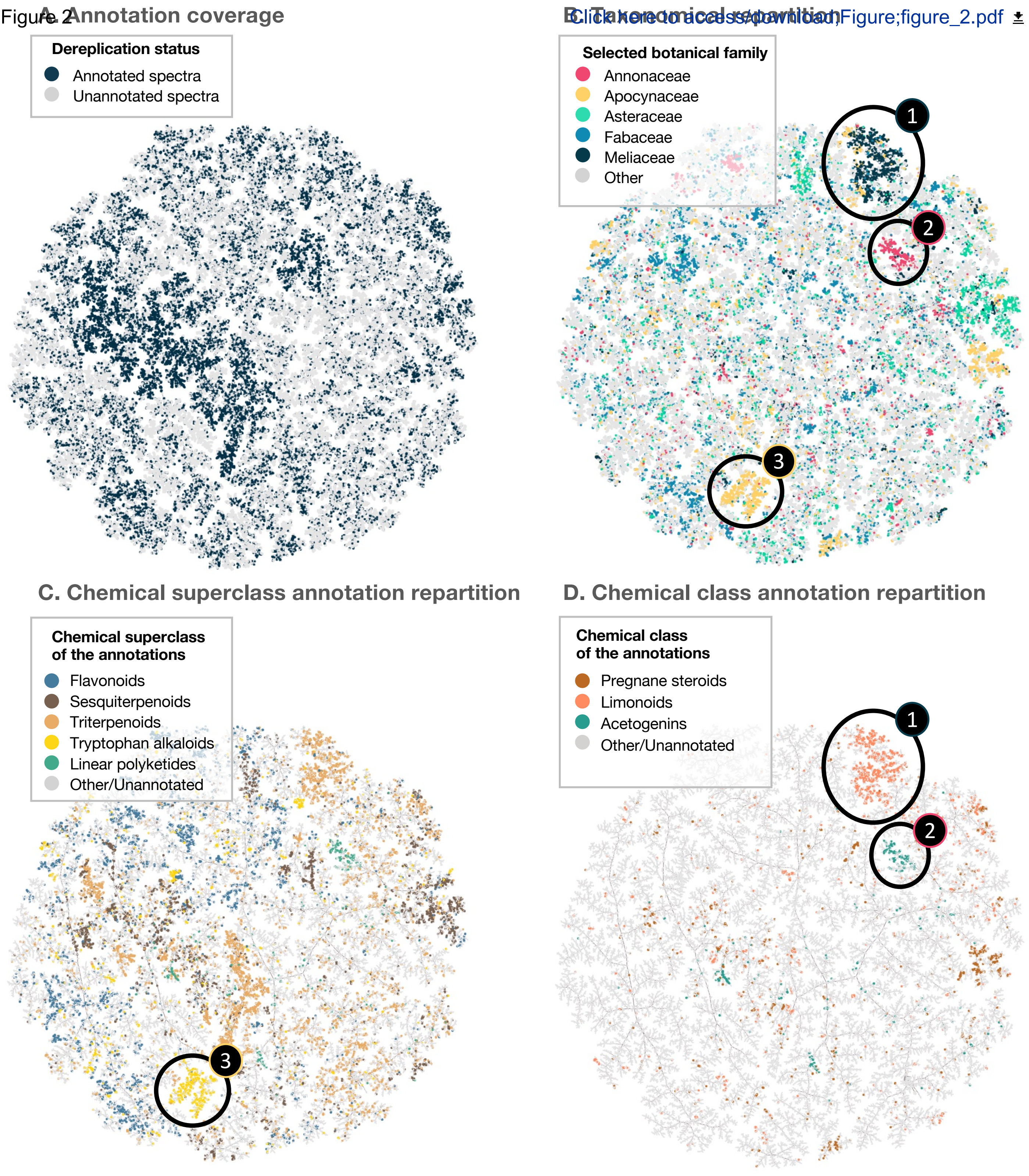

Figure 3

Click here to access/download;Figure;figure_3.pdf

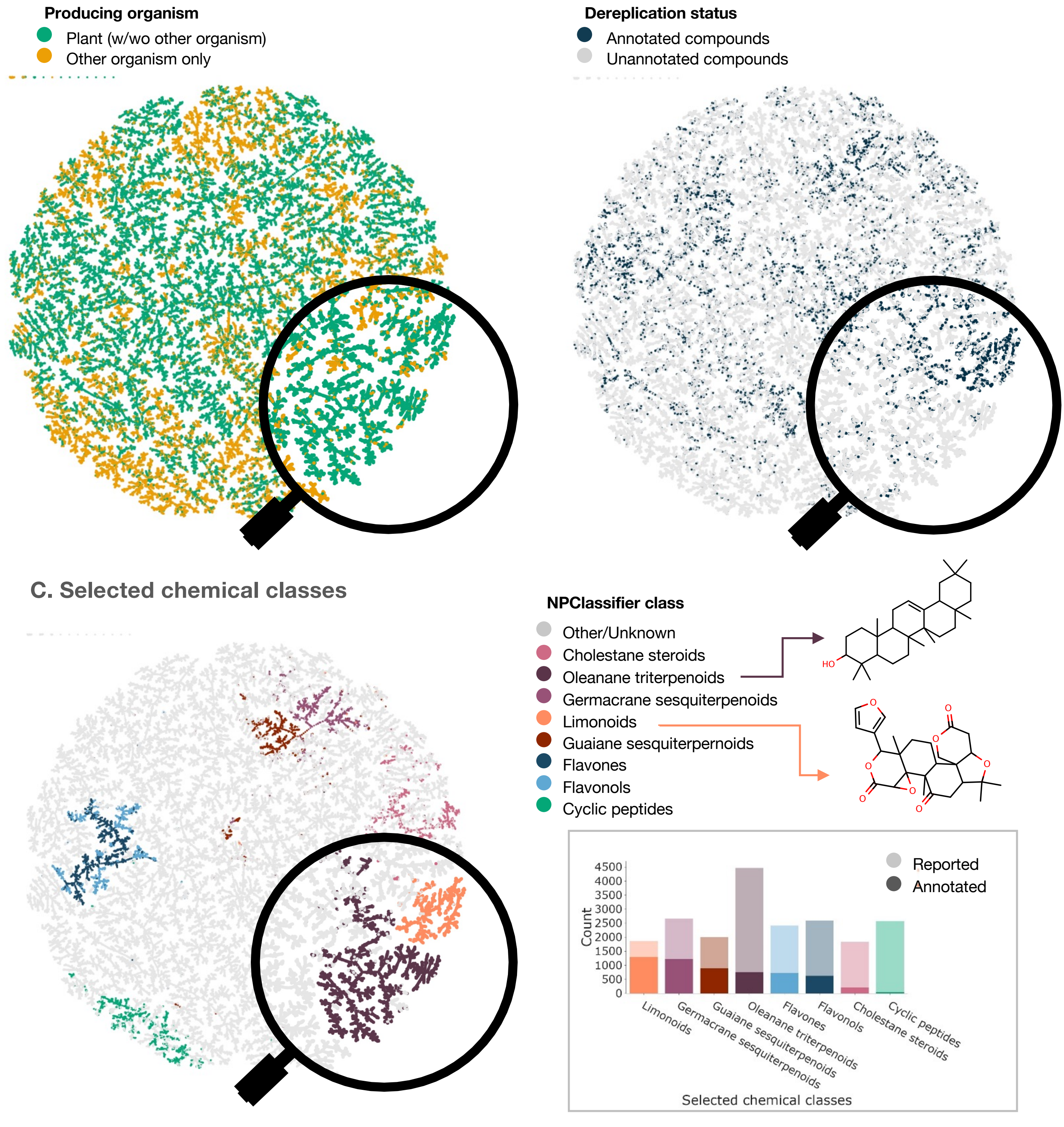

Supplement: giac124_GIGA-D-22-00126_Revision_1 [file giac124_giga-d-22-00126_revision_1.pdf]
