# Supplementary material for: Open and reusable annotated mass spectrometry dataset of a chemodiverse collection of 1,600 plant extracts
Source: Gigascience. 2023 Jan 18;12:giac124. doi: 10.1093/gigascience/giac124 (PMC9845059; doi:10.1093/gigascience/giac124)

GigaScience

Open and re-usable annotated mass spectrometry dataset of a chemodiverse collection of 1,600 plant extracts.

--Manuscript Draft--

**Manuscript Number:**	GIGA-D-22-00126R2
**Full Title:**	Open and re-usable annotated mass spectrometry dataset of a chemodiverse collection of 1,600 plant extracts.
**Article Type:**	Data Note
**Funding Information:**	Schweizerischer Nationalfonds zur Förderung der Wissenschaftlichen Forschung (CRSII5_189921/1) — Pr. Jean-Luc Wolfender
**Abstract:**	As privileged structures, natural products often display potent biological activities. However, the discovery of novel bioactive scaffolds is often hampered by the chemical complexity of the biological matrices they are found in. Large natural extracts collections are thus extremely valuable for their chemical novelty potential but also complicated to exploit in the frame of drug-discovery projects. In the end, it is the pure chemical substances that are desired for structural determination purposes and bioactivity evaluation. Researchers interested in the exploration of large and chemodiverse extracts collections should thus establish strategies aiming to efficiently tackle such chemical complexity and access these structures. Establishing carefully crafted digital layers documenting the spectral and chemical complexity as well as bioactivity results of natural products extracts collections can help to prioritize time-consuming but mandatory isolation efforts. In this note, we report the results of our initial exploration of a collection of 1,600 plant extracts in the frame of a drug discovery effort. After describing the taxonomic coverage of this collection, we present the results of its liquid chromatography high-resolution mass spectrometric profiling and the exploitation of these profiles using computational solutions. The resulting annotated mass spectral dataset and associated chemical and taxonomic metadata are made available to the community and data reuse cases are proposed. We are currently continuing our exploration of this plant extracts collection for drug-discovery purposes (notably looking for novel anti-trypanosomatids, anti-infective and prometabolic compounds) and eco-metabolomics insights. We believe that such a dataset can be exploited and reused by researchers interested in computational natural products exploration.
**Corresponding Author:**	Pierre-Marie Allard<br>University of Fribourg<br>Fribourg, Freiburg SWITZERLAND
**Corresponding Author Secondary Information:**	
**Corresponding Author's Institution:**	University of Fribourg
**Corresponding Author's Secondary Institution:**	
**First Author:**	Pierre-Marie Allard
**First Author Secondary Information:**	
**Order of Authors:**	Pierre-Marie Allard
	Arnaud Gaudry
	Luis-Manuel Quirós-Guerrero
	Adriano Rutz
	Miwa Dounoue-Kubo
	Tom W. N. Walker

Powered by Editorial Manager® and ProduXion Manager® from Aries Systems Corporation

	Emmanuel Defossez
	Christophe Long
	Antonio Grondin
	Bruno David
	Jean-Luc Wolfender
**Order of Authors Secondary Information:**	
**Response to Reviewers:**	Dear GigaScience editorial office,<br><br>We have integrated and responded to the formatiing comments on this last revised version.<br>Looking forward to read from you,<br><br>Best regards,<br><br>Pierre-Marie Allard
**Additional Information:**	
**Question**	**Response**
Are you submitting this manuscript to a special series or article collection?	No
**Experimental design and statistics**<br><br>Full details of the experimental design and statistical methods used should be given in the Methods section, as detailed in our Minimum Standards Reporting Checklist. Information essential to interpreting the data presented should be made available in the figure legends.<br><br>Have you included all the information requested in your manuscript?	No
If not, please give reasons for any omissions below.<br><br>as follow-up to "**Experimental design and statistics**<br><br>Full details of the experimental design and statistical methods used should be given in the Methods section, as detailed in our Minimum Standards Reporting Checklist. Information essential to interpreting the data presented should be made available in the figure legends.	Not applicable. Not statistical analysis in this datanote.

Powered by Editorial Manager® and ProduXion Manager® from Aries Systems Corporation

Have you included all the information requested in your manuscript?<br><br>"	
**Resources**<br><br>A description of all resources used, including antibodies, cell lines, animals and software tools, with enough information to allow them to be uniquely identified, should be included in the Methods section. Authors are strongly encouraged to cite Research Resource Identifiers (RRIDs) for antibodies, model organisms and tools, where possible.<br><br>Have you included the information requested as detailed in our Minimum Standards Reporting Checklist?	Yes
**Availability of data and materials**<br><br>All datasets and code on which the conclusions of the paper rely must be either included in your submission or deposited in publicly available repositories (where available and ethically appropriate), referencing such data using a unique identifier in the references and in the “Availability of Data and Materials” section of your manuscript.<br><br>Have you have met the above requirement as detailed in our Minimum Standards Reporting Checklist?	Yes

Powered by Editorial Manager® and ProduXion Manager® from Aries Systems Corporation

Manuscript

Click here to access/download;Manuscript;20221125_PF_datanote-

Open and re-usable annotated mass spectrometry dataset of a chemodiverse collection of 1,600 plant extracts.

Pierre-Marie Allard[1,2,3,*], pierre-marie.allard@unifr.ch, 0000-0003-3389-2191
Arnaud Gaudry[1,2], arnaud.gaudry@unige.ch, 0000-0002-3648-7362
Luis-Manuel Quirós-Guerrero[1,2], luis.guerrero@unige.ch, 0000-0002-1630-8697
Adriano Rutz[1,2], adriano.rutz@unige.ch, 0000-0003-0443-9902
Miwa Dounoue-Kubo[4], miwa-k@ph.bunri-u.ac.jp, 0000-0002-7271-3128
Tom W. N. Walker[5], thomas.walker@unine.ch, 0000-0001-8095-6026
Emmanuel Defossez[3,5], emmanuel.defossez@unifr.ch, 0000-0002-3279-9190
Christophe Long[6], christophe.long@pierre-fabre.com, 0000-0002-7677-7700
Antonio Grondin[7], antonio.grondin@pierre-fabre.com, 0000-0002-2952-6271
Bruno David[7], brunoxdavid@gmail.com, 0000-0002-6222-9228
Jean-Luc Wolfender[1,2], jean-luc.wolfender@unige.ch, 0000-0002-0125-952X

[1]Institute of Pharmaceutical Sciences of Western Switzerland, University of Geneva, 1211 Geneva 4, Switzerland
[2] School of Pharmaceutical Sciences, University of Geneva, 1211 Geneva 4, Switzerland
[3] Department of Biology, University of Fribourg, 1700 Fribourg, Switzerland
[4] Faculty of Pharmaceutical Sciences, Tokushima Bunri University, 180 Yamashiro-cho, Tokushima, 770-8514, Japan.
[5] Institute of Biology, University of Neuchâtel, 2000 Neuchâtel, Switzerland
[6] Direction Scientifique Naturactive, Pierre Fabre Medicament, 17 Avenue Jean Moulin, 81100 Castres, France.
[7] Green Mission Pierre Fabre, Institut de Recherche Pierre Fabre, 3 Avenue Hubert Curien, 31562 Toulouse, France.

* corresponding author

Abstract

As privileged structures, natural products often display potent biological activities. However, the discovery of novel bioactive scaffolds is often hampered by the chemical complexity of the biological matrices they are found in. Large natural extract collections are thus extremely valuable for their chemical novelty potential but also complicated to exploit in the frame of drug-discovery projects. In the end, it is the pure chemical substances that are desired for structural determination purposes and bioactivity evaluation. Researchers interested in the exploration of large and chemodiverse extract collections should thus establish strategies aiming to efficiently tackle such chemical complexity and access these structures. Establishing carefully crafted digital layers documenting the spectral and chemical complexity as well as bioactivity results of natural products extracts collections can help prioritize time-consuming but mandatory isolation efforts. In this note, we report the results of our initial exploration of a collection of 1,600 plant extracts in the frame of a drug discovery effort. After describing the taxonomic coverage of this collection, we present the results of its liquid chromatography high-resolution mass spectrometric profiling and the exploitation of these profiles using computational solutions. The resulting annotated mass spectral dataset and associated chemical and taxonomic metadata are made available to the community and data reuse cases are proposed. We are currently continuing our exploration of this plant extract collection for drug-discovery purposes (notably looking for novel anti-trypanosomatids, anti-infective and prometabolic compounds) and eco-metabolomics insights. We believe that such a dataset can be exploited and reused by researchers interested in computational natural products exploration.

Keywords

Plant extracts collection, metabolomics, drug discovery, LC-MS, natural products, mass spectrometry, chemodiversity, open science, biodiversity digitization

Data Description

In the frame of a partnership between academia and industry, a collaboration was established in 2017 between the Laboratory of Phytochemistry and Bioactive Natural Products at the University of Geneva (LPBNP), Switzerland, and the Pierre Fabre Laboratories (PFL) in Toulouse, France. This collaboration had the objective to explore and exploit the chemodiversity of a large collection of plant extracts (furnished by the industrial partner) using state-of-the-art mass spectrometric profiling methods and associated data mining solutions (performed and developed by the academic partner). The main goal of this research project is to establish a precise and exploitable description of the wide chemical diversity displayed in the plant extracts collection in order to orient further isolation efforts aiming to discover, in a first move, novel anti-trypanosomatids scaffolds and deepen chemotaxonomic knowledge of such a large set.

Context

Pierre Fabre Laboratories were founded in 1962 by Pierre Fabre, a French pharmacist from Castres (Tarn, France). They have specialized since the very beginning in the exploration and valorization of plants as medicines, health, and beauty products. Innovation based on the vegetal world in PFL encompasses chemotherapy treatments with vinca alkaloids extracted from the leaves of *Catharanthus roseus* (Q161093) [1], dermo-cosmetic products such as hair dyes from *Lawsonia inermis* (Q182448) extracts [2], or celastrol (Q5057534)-enriched extracts for psoriatic skin obtained from in vitro plant cell culture of *Tripterygium wilfordii* (Q1424919) [3]. In 1998, Pierre Fabre decided to launch a High Throughput Screening (HTS) program based on plant extracts with the objective to find novel anti-cancer drugs. The first samples of plant parts were collected in 1998 and when the HTS program ended in December 2015, the collection contained just over 17,000 unique samples. Multiple scientific articles originating from the exploration of this unique collection have been published over those years [4–7]. Hereafter is a selection of natural products-based scaffolds that entered antitumor medicinal chemistry programs: dimeric derivatives of artemisinin (Q426921) [8]; flavagline derivatives (Q3073444) [9]; griseofulvin (Q416096) [10]; narciclasine (Q18379239) and pancratistatin (Q7130395) [11]; neoboutomellerone [12]; triptolide (Q906351) [13].

The PFL collection, which is among the largest collection of plant samples worldwide with over 17,000 unique samples, was registered on April 1, 2020 at the European Commission under the accession number 03-FR-2020. This official registration recognizes the legality of the access and management process. It means that the collection meets the criteria set out in the European Access and Benefit Sharing (EU ABS) Regulation which implements at the European level the requirements of the

Nagoya Protocol regarding access to genetic resources and the fair and equitable sharing of benefits arising from their utilization [14]. To date three European collections are recognized [15].

In 2015 the Nature Open Library program was launched in order to share the unique PFL expertise with industrial or academic partners and to foster the research, development and industrialization of plant assets. In this context, PFL provided access to their private plants' collection, including some rare species [16]. Upon restructuration, this program was discontinued as of 2018. Some academic collaborations established at this time were kept active. The partnership established between LPBNP and PFL in 2017 had for principal objective the chemical characterization of the full PFL plant extracts collection. In order to evaluate the feasibility of such an ambitious program, a pilot project was defined. It focussed on a selection of 1,600 samples (corresponding to approximately 10% of the extracts prepared in the full PFL collection). The results of this pilot study, namely the generated data and outcomes are shared in this data note.

Note : from now on, in this manuscript, the full collection (17,000 extracts) is referred to as the "*PFL collection*". The selected set is simply referred to as "*the 1,600 extracts collection*".

Sample collection

All samples from the PFL collection were collected with respect to the different regulations around genetic resources at the time of collection. After drying and grounding, they were stored at room temperature, in the dark, in high-density polyethylene (HDPE) bar-coded 0.5L or 2L-pots. The storage room is access secured and protected by an automatic fire protection system with inert gas (50% nitrogen, 50% argon). Precise localization of the geographical sampling sites, unique IDs, barcodes and quantities, are stored in the PFL internal data management system. Furthermore, to perform later sample inspections, for example in case of dubious botanical identifications, all dry plant parts were also sampled and stored in their intact form. Several constraints were followed while building this collection. First, to have the best chances of finding new chemical entities, a large chemical diversity was desirable. Taking the assumption that taxonomical position and chemical production were related, samples were collected in order to maximize diversity within superior plant taxa (classes, orders, family and genera, see Figure 1). Second, only plants providing reasonable biomass quantities (i.e. > 50 g dry ground samples) were collected, hence allowing isolation of potentially active compounds in sufficient quantities to be characterized and to perform preliminary bioassays. Third, for preserving samples on the long term but also for allowing only stable compounds to be left in the samples, they

were dried for three days at 55°C. This point foresaw issues of potential HTS hits due to unstable compounds which would be difficult to isolate and manipulate.

The phylogenetic coverage of the 1,600 extracts collection is depicted in Figure 1. Within the Streptophyta (Q133527) phylum the collection represented ca. 64 % of the known orders and ca. 30 % of all known botanical families. The numbers rapidly decreased to ca. 4 % of the known genus and less than 0.5 % of all known species. See horizontal bar plot on Figure 1. The botanical families of the collection are relatively well distributed across the global phylogeny, despite some orders (e.g. Sapindales (Q26316), Rosales (Q21895)) being better covered than others (e.g. Crossosomatales (Q21860), Cyatheales (Q623232)). Interactive bar plots are available for the inspection of the coverage of orders (by families) and of families (by genus). The scripts for taxonomical names resolving and figure generation are available online [17].

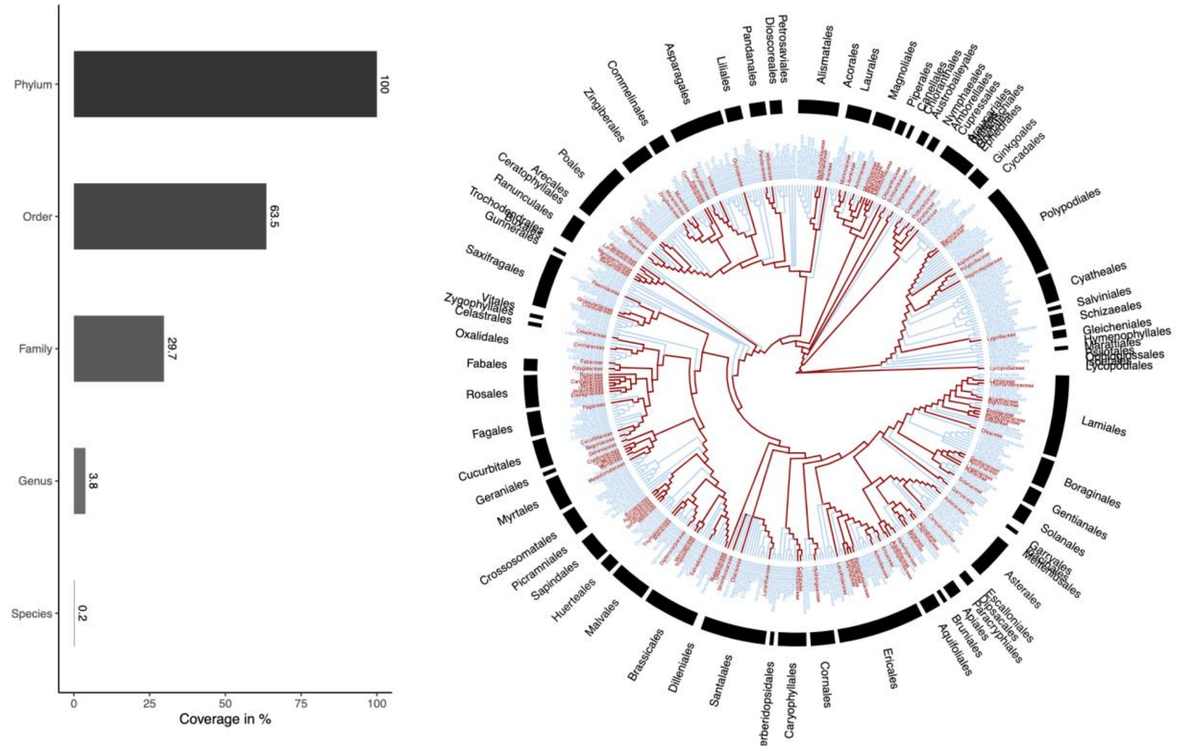

Figure 1: Taxonomical coverage of the profiled collection (1,600). On the left, the bar plot represents the overall coverage at main taxa level and up to the phylum Streptophyta. On the right, the taxonomical coverage is represented using a taxonomical tree of all families within the Streptophyta phylum. The families present within the current collection are highlighted in red. The Open Tree of Life (ott3.3) was used for taxonomy resolving. Download here.

Extraction and sample preparation

Since the PFL collection was designed to provide extracts for HTS experiments, only medium polarity compounds were targeted. Using ethyl acetate (EtOAc) extracts purified over silica gel allowed to reduce the amount of known classes of pan-assay interference compounds (PAINS) such as condensed tannins [18].

Starting from the dried plant material, the extracts were prepared as follows. A sample of 8 g (leaves, whole plant or aerial parts) or 10 g (subterranean parts, roots, bark) was extracted over a period of 8-10 h with 80 mL or 100 mL of EtOAc respectively. The extracts were filtered through paper and dried under vacuum in a rotary evaporator to a reduced volume (ca. 5 mL). The residue was transferred to a pre-weighted recipient and dried under vacuum until measurement of constant weight. The extracts were dissolved in EtOAc at a concentration of 30 mg/mL. An aliquot of 1 mL was mixed with 200 mg of silica gel (Merk 60, 40-63 μm) and dried under vacuum. The resulting powder mixture was transferred to a SPE cartridge (6 mL, 1 g SiO2). The cartridge was eluted with 10 mL of dichloromethane to remove apolar compounds, then the cartridge was eluted with 8 mL of a mixture dichloromethane:methanol (85:15), and subsequently washed with 2 mL of the same mixture to fully recover the compounds of interest. The filtrate (ca 10 mL final volume) was dried under vacuum. Extracts were then dissolved in DiMethyl SulfOxide (DMSO) to a concentration of 5 mg/mL and transferred to 1 mL 96 deep-well plates for further analysis. For Liquid Chromatography hyphenated to Mass Spectrometry (LC-MS) analysis (see below), the extracts were dissolved in DMSO to reach a concentration of 2.5 mg/mL. The plates originally contained 80 extracts per plate, the columns 1 and 12 being empty. These columns were filled, respectively, by a QC (quality control) sample and a DMSO blank. The QC samples were constituted by a mixture of *Cinchona pubescens* Vahl (Q164574), *Panax ginseng* C.A.Mey. (Q182881), *Ginkgo biloba* L. (Q43284), *Arnica montana* L. (Q207848) and *Salvia officinalis* L. (Q1111359) methanolic extracts dissolved in a 1:1:1:1:1 ratio at 5 mg/mL in DMSO. These blanks and QC samples were injected every ten samples.

LC-MS/MS analyses

Chromatographic separation was performed on a Waters Acquity UPLC system interfaced to a Q-Exactive Focus mass spectrometer (Thermo Scientific, Bremen, Germany), using a heated electrospray ionization (HESI-II) source. Thermo Scientific Xcalibur 3.1 software was used for instrument control. The LC conditions were as

follows: column, Waters BEH C18 50 × 2.1 mm, 1.7 μm; mobile phase, (A) water with 0.1% formic acid; (B) acetonitrile with 0.1% formic acid; flow rate, 600 μL·min^{-1}; injection volume, 2 μL; gradient, linear gradient of 5−100% B over 7 min and isocratic at 100% B for 1 min. The optimized HESI−II parameters were as follows: source voltage, 3.5 kV (pos); sheath gas flow rate (N2), 55 units; auxiliary gas flow rate, 15 units; spare gas flow rate, 3.0; capillary temperature, 350.00°C, S-Lens RF Level, 45. The mass analyzer was calibrated using a mixture of caffeine, methionine–arginine–phenylalanine–alanine–acetate (MRFA), sodium dodecyl sulfate, sodium taurocholate, and Ultramark 1621 in an acetonitrile/methanol/water solution containing 1% formic acid by direct injection. The data-dependent MS/MS events were performed on the three most intense ions detected in full scan MS (Top3 experiment). The MS/MS isolation window width was 1 Da, and the stepped normalized collision energy (NCE) was set to 15, 30 and 45 units. In data-dependent MS/MS experiments, full scans were acquired at a resolution of 35,000 Full Width at Half Maximum (FWHM) (at *m/z* 200) and MS/MS scans at 17,500 FWHM both with an automatically determined maximum injection time. After being acquired in a MS/MS scan, parent ions were placed in a dynamic exclusion list for 2.0 s.

Data-processing and Molecular Networking

The MS data were converted from .RAW (Thermo) standard data format to .mzXML format using the MSConvert software, part of the ProteoWizard package [19]. The converted files were treated using the MZMine software suite v. 2.53 [20]. The parameters were adjusted as follows: the centroid mass detector was used for mass detection with the noise level set to 1.0E4 for MS level set to 1, and to 0 for MS level set to 2. The ADAP chromatogram builder was used and set to a minimum group size of scans of 5, minimum group intensity threshold of 1.0E4, minimum highest intensity of 5.0E5 and *m/z* tolerance of 12 ppm [21]. For chromatogram deconvolution, the algorithm used was the wavelets (ADAP). The intensity window S/N was used as S/N estimator with a signal to noise ratio set at 10, a minimum feature height at 5.0E5, a coefficient area threshold at 130, a peak duration ranges from 0.0 to 0.5 min and the RT wavelet range from 0.01 to 0.03 min. Isotopes were detected using the isotopes peaks grouper with a *m/z* tolerance of 12 ppm, a RT tolerance of 0.01 min (absolute), the maximum charge set at 2 and the representative isotope used was the most intense. Each feature list was filtered before alignment to keep only features with an associated MS2 scan and a RT between 0.5 and 8.0 min using the feature filtering. Peak alignment was performed using the join aligner method (*m/z* tolerance at 40 ppm), absolute RT tolerance 0.2 min, weight for *m/z* at 2 and weight for RT at 1 and a weighted dot-product cosine similarity of 0.3. The aligned feature list (119,182 features) was exported using

the export to GNPS module. Features occurring in QC samples only or in blanks were removed before molecular networking, resulting in a final feature list of 117,005 features. The MZMine parameters used for the data treatment are available at the referenced link [22] .

The initial study of this aligned peak list with classical unsupervised statistics (e.g. Principal Component Analysis [PCA], Principal Coordinates Analysis [PCoA]) indicated a strong batch effect which could be tracked down to a specific date and attributed to a change of column during the course of the mass spectrometry analysis of the full collection. Such batch effects are almost inevitable when samples are profiled on long periods (here spanning over several months) and are particularly complicated to mitigate especially when dealing with chemodiverse datasets where poor overlap among the samples is expected. This incentivized us to develop a novel computational mass spectrometry solution for MS2 BasEd SaMple VectOrization (MEMO). MEMO allows to organize samples in large and chemodiverse collections in a retention-time agonist fashion thus strongly mitigating batch effect and allowing the comparison of samples acquired over heterogeneous chromatographic conditions. Applied to the current plant extract collection, MEMO allows efficient reduction of the observable batch-effect and clustering samples according to their content [23]. See the referenced link for a view of the batch effect on the classically aligned dataset and its mitigation using MEMO fingerprints [24].

To analyze the spectral diversity of the profile collection, a molecular network (MN) was created on the GNPS website (http://gnps.ucsd.edu) using the .mgf spectra file generated at the previous step [25]. The precursor ion mass tolerance was set to 0.02 Da and a MS/MS fragment ion tolerance of 0.02 Da. A network was then created where edges were filtered to have a cosine score above 0.7 and more than 6 matched peaks. Further, edges between two nodes were kept in the network if and only if each of the nodes appeared in each other's respective top 10 most similar nodes. Finally, the maximum size of a spectral family was set to 100, and the lowest scoring edges were removed from molecular families until the molecular family size was below this threshold. The spectra in the network were then searched against GNPS spectral libraries. All matches kept between network spectra and library spectra were required to have a score above 0.7 and at least 6 matched peaks. The resulting molecular network is available online [26]. A Cytoscape (RRID:SCR_003032) file corresponding to the full molecular network mapped with a color layout corresponding to NPClassifier chemical classification, the experimental and theoretical spectral matches as well as a feature table grouped at the family level is available through the following MassIVE repository link.

Via the GNPS Explorer interface it is possible to efficiently navigate through the uploaded spectral file and their associated metadata. See the referenced link [27]. For example, the name of a plant of interest can be typed under the NCBI Taxonomy header resulting in the direct filtering of the dataset for this specific plant. Individual spectral files can then be selected (and eventually compared if multiple are selected) and viewed using the GNPS dashboard. See for example this interactive view of the total ion chromatogram of *Desmodium heterophyllum* Hook. & Arn (Q10770714) aerial parts.

In addition to the MN, spectra (from the same .mgf file used for MN) were organized using TMAP visualization [28]. In this case the TMAP corresponds to a minimum spanning tree built from a dense network of spectral similarity. For the establishment of this visualization, spectra were first translated to documents (two decimals were used, i.e. a peak at 100.3897 would be translated as "peak@100.39") using matchms and spec2vec packages [29,30], with calculation of neutral losses (up to 400 *m/z*) to the precursor. The spectral documents, i.e. a list of peaks and losses without intensity information, were then hashed using the MinHash scheme and indexed in an locality-sensitive hashing (LSH) forest that was used to generate the TMAP visualization based on the presence/absence of peaks and losses in spectra [28] [31]. The generated TMAP is presented in Figure 2, an interactive visualization is available [32], and the code used for the TMAP generation is available online [17]. Such visualization allows to highlight taxa-specific spectral subspaces. In addition, structural annotation (see next section for details concerning the metabolite annotation process) were overlaid to their corresponding spectra. When no annotations were reported a NanoPutian was depicted as a placeholder. The structural annotations were limited to compounds reported in the Eukaryote domain. For example, the spectra region 1 in Figure 2 B. and 2 D. is mainly specific to samples belonging to the Meliaceae (Q158979) family and most of these spectra are annotated as limonoids derivatives (Q669514). Another region, called region 2 in Figure 2, contains mainly spectra specific to the Annonaceae (Q220025) family and corresponds to acetogenins derivatives (Q3604300). Finally, the spectral region 3 is specific to the Apocynaceae family (Q173756) and is mainly annotated as tryptophan alkaloids derivatives. On the other hand, botanical families such as the Fabaceae (Q44448) appear to occupy a much wider spectral space. These examples showcase the importance of the taxonomic coverage of an extract collection to maximize the chemical diversity. Indeed, if some chemical classes, such as the flavonoid (Q3561192) derivatives, appear to be widespread, some others are known to be extremely specific to some taxa, such as the acetogenins to the Annonaceae [33]. Of course, the interpretation of this spectral TMAP is highly dependent on the structural annotations' quality (see next section "Metabolite annotation") and as such should be taken with caution. Because the annotation process favored structures reported in closer taxa, it should also be noted that the comparison of annotation occurrences and taxonomy may

be impacted by an annotation set biased towards denser taxa-specific ensembles of structures.

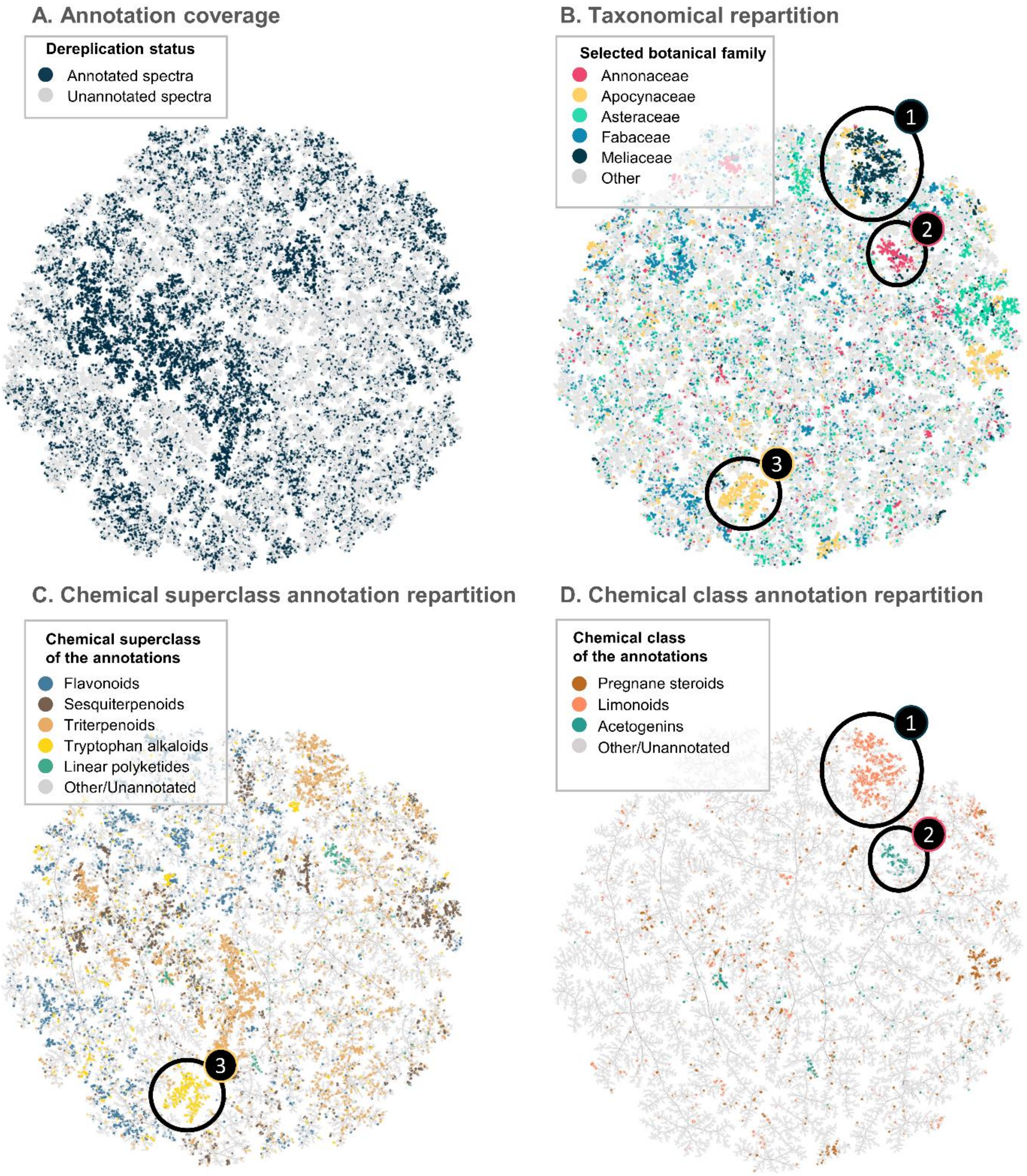

Figure 2: Spectral diversity of the profiled plant collection (1,600 extracts). The TMAP approach is employed to display the >100,000 of spectra resulting from the alignment of the 1,600 untargeted MS/MS experiments. In this TMAP each dot represents a feature's spectrum and they are linked together according to their similarity. In **A**, blue dots (36% of the total amount of spectra) correspond to annotated spectra while gray dots (64%) correspond to unannotated spectra. In **B**, dots are colored according to the botanical family of the sample where the highest MS1 peak area for the corresponding feature was recorded. In **C** and **D**, dots are colored according to the NPClassifier superclass and class, respectively, of their annotation (for annotated dots). In **B**, it is possible to spot spectral regions (1, 2 and 3) specific to given botanical families. The region 1 is specific to the Meliaceae family and these spectra are mainly annotated as limonoids derivatives, the region 2 is specific to the Annonaceae family and spectra are mainly annotated as acetogenins derivatives and the region 3 is specific to the Apocynaceae family and spectra are annotated as tryptophan alkaloids derivatives. Note that the structural annotation results are reweighed according to the taxonomical proximity of the biological source of the candidate structure and the biological source of the annotated spectra. A bias toward taxa-specific structures can thus be observed. This interactive structural TMAP can be browsed online [32].

Metabolite annotation

Experimental spectral libraries search

The full spectral dataset corresponding to the 1,600 plant extracts collection has been uploaded on the MASSIVE repository [34] https://doi.org/doi:10.25345/C59J97, a continuous identification workflow is automatically carried against GNPS experimental spectral libraries. The latest results of this continuous identification workflow can be observed online [35]. The latest iteration at the time of publication (2022-04-18) indicated that a total of 2665 unique compounds were spectrally matched [36].

Theoretical spectral libraries search

In addition to experimental spectral libraries search we have shown that spectral matching against theoretical spectral libraries of natural products was an efficient way to cover a much wider, yet relevant, spectral space [37]. Furthermore, we showed that taking into account the taxonomical distance between the biological source of candidate structure and the biological source of the annotated extracts greatly improved the overall quality of the annotation results [38]. Thus, in addition to the spectral search performed at the molecular networking step against publicly available spectral libraries (see previous section) a taxonomically informed metabolite annotation was performed. For this we first established a large theoretical spectral database of natural products following a previously established metabolite annotation workflow. This spectral database and associated biological sources metadata were constructed using chemical structure and information compiled during the LOTUS Initiative's first project aiming to establish an open and evolutive resource compiling natural products biological

occurrences [39]. The theoretical spectral database is publicly available [40]. The biological sources metadata are available online [41]. The taxonomically informed metabolite annotation was performed using the met_annot_enhancer scripts [42]. The parameters used for the taxonomically informed metabolite annotation process and the resulting tables can be found online [43]. PF_full_datanote_spectral_match_results_repond.tsv corresponds to the Cytoscape formatted output.

Visualization of the metabolite annotations

In order to obtain an overview of the metabolite annotation results on the profiled collection, these were compared to the ensemble of molecules described in LOTUS, See Figure 3. For this, a TMAP [28,44,45] was built to connect similar chemical structures using the MAP4 (MinHashed Atom-Pair fingerprint up to four bonds) fingerprint [46], the retrieval of the nearest neighbors was achieved as described above for the spectral TMAP construction. In Figure 3, each dot represents a chemical structure and it is linked to its neighbor according to its structural proximity. In Figure 3 A., the color code indicates the repartition of chemical structures mostly found to be present at least once in plants (green color) versus structures found only in other kingdoms (orange color). In Figure 3 B. each of the annotated structures within the 1,600 plants extracts collection is displayed (strong blue) within the rest of the reported structures in LOTUS (light gray) indicating a relatively heterogeneous structural coverage of the annotations with some denser areas. Finally, the color coding in Figure 3 C. allows to distinguish chemical classes in the overall TMAP. The NPClassifier classification is employed [47]. The framed bar plot displays the most frequently annotated chemical classes in the present dataset (strong color) versus the total count for each class in LOTUS (light color). Multiple factors can explain discrepancies between the repartition of chemical classes annotated in the 1,600 extracts collection versus the overall repetition observed in LOTUS. For example cyclic peptides (green bar in the framed bar plot) are poorly covered. This can be explained by the fact that such structures are most often found in microbial organisms (as observed when checking this region in plot A.). On the other hand, the high coverage of limonoids in the collection can be explained by the high proportion of species from the Sapindales order (see Figure 1. and this plot) and the fact that this order is known to be the main responsible of the biosynthesis of such scaffolds [48]. An interactive version of the structural TMAP presented in Figure 3 is available online [49].

A detailed coverage of annotations of the dataset versus the full LOTUS annotations is available as interactive plots (coverage at the pathway level, at the superclass level and at the class level.)

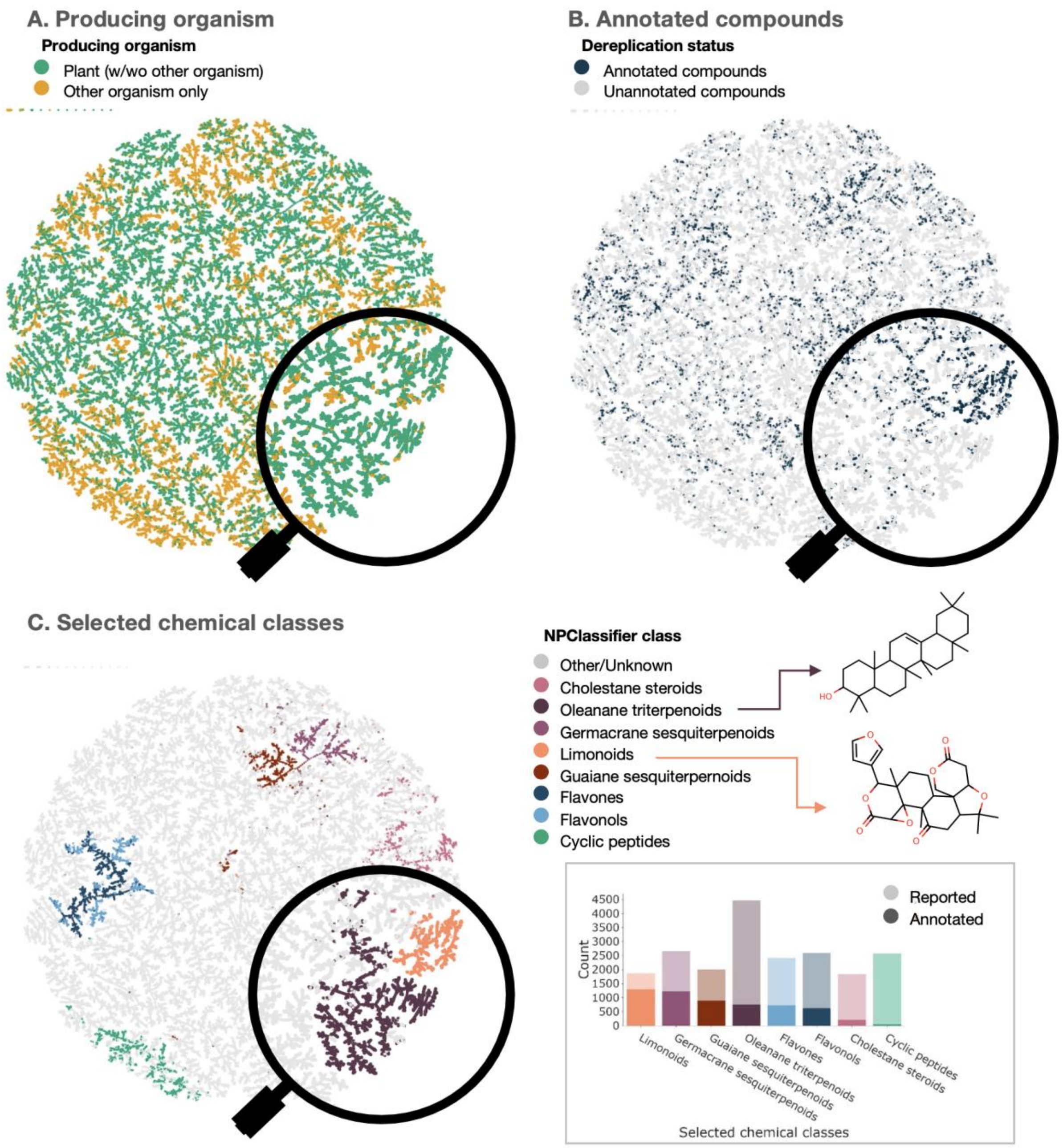

Figure 3: Chemical diversity of the profiled plant collection (1,600 extracts) and coverage against reported natural products. Visualization of reported natural products structures (LOTUS v1 and Dictionary of Natural Products v29.1) as a TMAP with plotting of the producing organism (A), the annotation's status, *i.e.* whether the 2D structure was annotated in the dataset (B) and selected NPClassifier classes (C). In the insert of C, the bar plot represents the number of compounds reported for each of the selected chemical classes with the opaque part of the bar representing the annotated compounds. The zoom on the limonoids and oleanane terpenoids clusters of the TMAP allows visualizing a well covered chemical class such as limonoids and a less covered one such as oleanane triterpenoids. A specific member of each class (limonin [Q2398745] for the limonoids and β-amyrin [Q27108621] for the oleanane triterpenoids) are represented in their planar structure form for illustration purposes. This interactive structural TMAP can be browsed online [49].

Reusability

Regarding data reuse, multiple options exist for such a dataset. Hereafter we describe some selected use cases. For example, GNPS continuous identification can be explored and retrieved online [50] and can be used to compare or combine the results of experimental library matches to theoretical spectral library matches. Since the dataset is also available through the ReDu interface, multiple reanalysis possibilities can be explored. For example, through the https://redu.ucsd.edu/metadataselection interface and after selection of the PF dataset by its DatasetAccession value (MSV000087728), a filter for all files of the dataset can be established. Filters of individual species can be obtained by selecting their corresponding NCBITaxonomy terms. Later on, the full set or subset thereof, can be selected for Molecular Networking or Library Search at GNPS for example. See the complete ReDu documentation for details [51].

The recently developed Mass Spec Query Language (MassQL) allows efficient search for specific patterns within large spectral datasets[52]. For example researchers can look for ionization patterns characteristic of dimeric monoterpene indole alkaloids (MIA) using the following MassQL query. The results of the query on the 1,600 extracts collection is available here. As an example, this query returns that a feature with a mass of 687.3570 corresponds to the searched criteria. This page displays the overlap of the MassQL query criteria and the corresponding MS1 and MS2 spectra, and also indicates that this spectra was found in sample VGF152_B02_pos.mzXML. We can then turn to the GNPS Explorer interface for the 1,600 plants dataset, filter for the sample id, and see that this sample is an extract of *Tabernaemontana coffeoides* (Q15376858) which is known to contain dimeric MIA. In the GNPS Explorer interface we can quickly filter for other species of *Tabernaemontana* (one is present, *Tabernaemontana crassa* (Q14862275)) and use the GNPS LCMS Dashboard (see plots) to observe that the incriminated feature is mostly present in *Tabernaemontana coffeoides.* A MassQL compendium groups query examples, these can be used to further explore the 1,600 extracts collection.

Conclusions

Here we presented the results of state-of-the-art computational approaches used for both spectral organization and metabolite annotation of high-resolution mass spectrometry data acquired on a large collection of 1,600 chemically diverse plant extracts. All original mass spectrometry profiles and associated metadata are made available to the community for further (re)analysis. The results of the metabolite annotation define a putative chemical space that can be exploited and refined in subsequent drug discovery,

chemotaxonomic or eco-metabolomics projects. Here we illustrate that, through partnerships between academia and industry, the faith of historical and private collections of plant extracts can be changed and the richness of the associated chemical diversity can be made available to a wider public.

Data availability

All .RAW (Thermo), .mzXML and .mzML datafiles in positive and negative ionization modes along with metadata and metabolite annotation tables are available on the MassIVE repository under accession number MSV000087728 [34]. Molecular networking job results are publicly available [53]. Furthermore, all supporting data and materials are also available in the *GigaScience* GigaDB database [54].

Availability of source code and requirements

The scripts used to generates the figures of this data Note are available as follows.

Project name: Scripts for the PF1600 project

Project home page: https://github.com/mandelbrot-project/pf_1600_datanote

Operating system: Platform independent

Programming language: R and Python

License: GNU Affero General Public License v3.0

Declarations

List of abbreviations

DMSO : DiMethyl SulfOxide
EU ABS : European Access and Benefit Sharing
EtOAc : ethyl acetate
FWHM : Full Width at Half Maximum
HDPE : high-density polyethylene
HESI-II : heated electrospray ionization
HTS : High Throughput Screening
LC-MS : Liquid Chromatography hyphenated to Mass Spectrometry
LPBNP : Laboratory of Phytochemistry and Bioactive Natural Products
LSH : locality-sensitive hashing
MAP4 : MinHashed Atom-Pair fingerprint up to four bonds
MassQL : Mass Spec Query Language
MEMO : MS2 BasEd SaMple VectOrization
MN : molecular network
MRFA : methionine–arginine–phenylalanine–alanine–acetate
NCE : normalized collision energy
PAINS : pan-assay interference compounds
PCA : Principal Component Analysis
PCoA : Principal Coordinates Analysis
PFL : Pierre Fabre Laboratories
QC : quality control
TMAP : Tree MAP

UMAP : uniform manifold approximation and projection

Competing interests

The authors declare that they have no competing interests

Funding

J-LW and P-MA are thankful to the Swiss National Science Foundation for the funding of the project (SNF N° CRSII5_189921/1).

Authors' contributions

Following the CRediT (Contributor Roles Taxonomy) roles.

Conceptualisation P-MA, BD and J-LW. Data curation P-MA, AGa, LQG, AR and MDK. Funding acquisition P-MA and J-LW. Investigation P-MA and MDK. Methodology P-MA, MDK and J-LW. Project administration P-MA, BD and J-LW. Resources CL, AGr, BD and J-LW. Software P-MA, AGa, TW, ED and AR. Supervision P-MA and J-LW. Validation P-MA, AGa and MDK. Visualization P-MA, AGa, ED and AR. Writing – original draft P-MA. Writing – review & editing P-MA, AGa, LQG, AR and AGr.

Acknowledgements

The authors are grateful to Green Mission Pierre Fabre, Pierre Fabre Research Institute, Toulouse, France for establishing and sharing this unique library of extracts.

References

1. Duflos A, Kruczynski A, Barret J-M. Novel aspects of natural and modified vinca alkaloids. *Curr Med Chem Anticancer Agents*. 2:55–702002;

2. Fiorini-Puybaret C, Joulia P. Dye composition comprising a combination of two plant extracts of Lawsonia inermis. World Patent WO2020249748A1;

3. Nguyen T, Cousy A, Steward N. Method for producing celastrol and pentacyclic triterpene derivatives. World Patent WO2017194757A1;

4. Vandenberghe I, Créancier L, Vispé S, Annereau J-P, Barret J-M, Pouny I, et al.. Physalin B, a novel inhibitor of the ubiquitin-proteasome pathway, triggers NOXA-associated apoptosis. *Biochem Pharmacol*. 76:453–622008;

5. Pouny I, Long C, Batut M, Aussagues Y, Jean Valère N, Achoundong G, et al.. Quinolizidine Alkaloids from Cylicomorpha solmsii. *J Nat Prod*. 84:1198–2022021;

6. Long C, Sauleau P, David B, Lavaud C, Cassabois V, Ausseil F, et al.. Bioactive flavonoids of Tanacetum parthenium revisited. *Phytochemistry*. 64:567–92003;

7. Pouny I, Batut M, Vendier L, David B, Yi S, Sautel F, et al.. Cytisine-like alkaloids from Ormosia hosiei Hemsl. & E.H. Wilson. *Phytochemistry*. 107:97–1012014;

8. Begue J-P, Bonnet-Delpon D, Crousse B, Fournial A, Mordant C, Fahy J. Dimeric derivatives of artemisinin and application in anti-cancer therapy. World Patent WO2010012761A1;

9. Marion F, Kaloun EB, Lieby-Muller F, Perez M, Annereau J-P, Creancier L. Flavagline derivatives. World Patent WO2016001441A1;

10. Marion F, Lieby-Muller F, Grisoni S, Rahier N, Perez M, Sartori I. Griseofulvin derivatives. World Patent WO2014020101A1;

11. Marion F, Annereau J-P, Fahy J. Nitrogenated derivatives of pancratistatin. World Patent WO2010012714A1;

12. Beck J, Guminski Y, Long C, Marcourt L, Derguini F, Plisson F, et al.. Semisynthetic neoboutomellerone derivatives as ubiquitin-proteasome pathway inhibitors. *Bioorg Med Chem*. 20:819–312012;

13. Kaloun EB, Long C, Molinier N, Brel V, Cantagrel F, Massiot G. Partial synthesis of 14-deoxy-14-aminotriptolide. *Tetrahedron Lett*. 57:1895–82016;

14. EUR-Lex - 32014R0511 - EN - EUR-Lex.

http://data.europa.eu/eli/reg/2014/511/oj Accessed 2022 Nov 12.

15. Register of Collections - EU ABS Regulation. https://ec.europa.eu/environment/nature/biodiversity/international/abs/pdf/Register%20of%20Collections.pdf Accessed 2022 Nov 11.

16. Pierre Fabre laboratories unveils Open Nature Library, a unique open innovation program in the world. https://www.pierre-fabre.com/en/press_release/pierre-fabre-laboratories-unveils-open-nature-library-a-unique-open-innovation Accessed 2022 Nov 11.

17. pf_1600_datanote scripts. GitHub. https://github.com/mandelbrot-project/pf_1600_datanote/releases/tag/v0.1 Accessed 2022 Nov 11.

18. David B, Ausseil F. High-Throughput Screening of Plant Chemodiversity. Encyclopedia of Analytical Chemistry. https://doi.org/10.1002/9780470027318.a9944

19. Chambers MC, Maclean B, Burke R, Amodei D, Ruderman DL, Neumann S, et al.. A cross-platform toolkit for mass spectrometry and proteomics. *Nat Biotechnol*. 30:918–202012;

20. Pluskal T, Castillo S, Villar-Briones A, Orešič M. MZmine 2: Modular framework for processing, visualizing, and analyzing mass spectrometry-based molecular profile data. *BMC Bioinformatics*. 11:3952010;

21. Myers OD, Sumner SJ, Li S, Barnes S, Du X. One Step Forward for Reducing False Positive and False Negative Compound Identifications from Mass Spectrometry Metabolomics Data: New Algorithms for Constructing Extracted Ion Chromatograms and Detecting Chromatographic Peaks. *Anal Chem*. 89:8696–7032017;

22. 210302_VGF_pos_parameters.xml. https://massive.ucsd.edu/ProteoSAFe/DownloadResultFile?file=f.MSV000087728/updates/2022-05-02_pmallard_e88304cd/other/210302_VGF_pos_parameters.xml Accessed 2022 Nov 11.

23. Gaudry A, Huber F, Nothias L-F, Cretton S, Kaiser M, Wolfender J-L, et al.. MEMO: Mass Spectrometry-Based Sample Vectorization to Explore Chemodiverse Datasets. *Front Bioinform*. Frontiers Media SA; 2022; doi: 10.3389/fbinf.2022.842964.

24. Comparative PCoA with samples colored according to their injection date (2 groups). https://mandelbrot-project.github.io/memo_publication_examples/plant_extract_dataset/pcoa_vgf_color_before_after.html Accessed 2022 Nov 11.

25. Wang M, Carver JJ, Phelan VV, Sanchez LM, Garg N, Peng Y, et al.. Sharing and community curation of mass spectrometry data with GNPS. *Nat Biotechnol*. 34:828–372016;

26. UCSD Computational Mass Spectrometry Website. https://proteomics2.ucsd.edu/ProteoSAFe/status.jsp?task=3197f70bed224f9ba6f59f62906839e9 Accessed 2022 Nov 11.

27. GNPS - Dataset Browser. https://gnps-explorer.ucsd.edu/MSV000087728?dataset_accession=MSV000087728&metadata_source=MASSIVE&metadata_option=f.MSV000087728%2Fupdates%2F2021-11-15_pmallard_334e9199%2Fmetadata%2Fgnps_metadata.tsv Accessed 2022 Nov 11.

28. Probst D, Reymond J-L. Visualization of very large high-dimensional data sets as minimum spanning trees. *J Cheminform*. 12:122020;

29. Huber F, Verhoeven S, Meijer C, Spreeuw H, Castilla E, Geng C, et al.. Matchms - processing and similarity evaluation of mass spectrometry data. *J Open Source Softw*. The Open Journal; 5:24112020;

30. Huber F, Ridder L, Verhoeven S, Spaaks JH, Diblen F, Rogers S, et al.. Spec2Vec: Improved mass spectral similarity scoring through learning of structural relationships. *PLoS Comput Biol*. 17:e10087242021;

31. LSH forest: self-tuning indexes for similarity search. ACM Digital Library. https://doi.org/10.1145/1060745.1060840 Accessed 2022 Nov 12.

32. pf1600_spectral_tmap_pos. https://mandelbrot-project.github.io/pf_1600_datanote/data/outputs/spectral/tmap/pf1600_spectral_tmap_pos.html Accessed 2022 Nov 11.

33. Neske A, Ruiz Hidalgo J, Cabedo N, Cortes D. Acetogenins from Annonaceae family. Their potential biological applications. *Phytochemistry*. 174:1123322020;

34. Allard P-M and Wolfender J-L. MassIVE MSV000087728 - GNPS_PF_plant_extracts_library_dataset_01. MassIVE 2021. https://doi.org/10.25345/c59j97

35. UCSD Computational Mass Spectrometry Website. https://gnps.ucsd.edu/ProteoSAFe/result.jsp?task=b753bf1e39cb4875bdf3b786e747bc15&view=advanced_view Accessed 2022 Nov 11.

36. UCSD Computational Mass Spectrometry Website. https://gnps.ucsd.edu/ProteoSAFe/result.jsp?task=ee2e8e9cf8214f48ab6ee01df652a3f2&view=all_unique_compounds Accessed 2022 Nov 11.

37. Allard P-M, Péresse T, Bisson J, Gindro K, Marcourt L, Pham VC, et al.. Integration of Molecular Networking and In-Silico MS/MS Fragmentation for Natural Products Dereplication. *Anal Chem*. 88:3317–232016;

38. Rutz A, Dounoue-Kubo M, Ollivier S, Bisson J, Bagheri M, Saesong T, et al.. Taxonomically Informed Scoring Enhances Confidence in Natural Products

Annotation. *Front Plant Sci*. 10:13292019;

39. Rutz A, Sorokina M, Galgonek J, Mietchen D, Willighagen E, Gaudry A, et al.. The LOTUS initiative for open knowledge management in natural products research. *eLife*. 2022; doi: 10.7554/eLife.70780.

40. Allard P-M, Bisson J, Rutz A. ISDB: In Silico Spectral Databases of Natural Products; Zenodo 2021 http://dx.doi.org/10.5281/zenodo.5607264

41. Rutz A, Bisson J, Allard P-M. The LOTUS Initiative for Open Natural Products Research: frozen dataset union wikidata (with metadata); Zenodo 2022 http://dx.doi.org/10.5281/zenodo.6378204

42. met_annot_enhancer v0.1. GitHub. https://github.com/mandelbrot-project/met_annot_enhancer/releases/tag/v0.1 Accessed 2022 Nov 11.

43. MassIVE Dataset Files. https://massive.ucsd.edu/ProteoSAFe/dataset_files.jsp?task=b753bf1e39cb4875bdf3b786e747bc15#%7B%22table_sort_history%22%3A%22main.collection_asc%22%2C%22main.attachment_input%22%3A%22updates%2F2022-04-28_pmallard_b0e0f70c%22%7D Accessed 2022 Nov 11.

44. Probst D, Reymond J-L. SmilesDrawer: Parsing and Drawing SMILES-Encoded Molecular Structures Using Client-Side JavaScript. *J Chem Inf Model*. 58:1–72018;

45. Probst D, Reymond J-L. FUn: a framework for interactive visualizations of large, high-dimensional datasets on the web. *Bioinformatics*. 34:1433–52018;

46. Capecchi A, Probst D, Reymond J-L. One molecular fingerprint to rule them all: drugs, biomolecules, and the metabolome. *J Cheminform*. 12:432020;

47. Kim HW, Wang M, Leber CA, Nothias L-F, Reher R, Kang KB, et al.. NPClassifier: A Deep Neural Network-Based Structural Classification Tool for Natural Products. *J Nat Prod*. 84:2795–8072021;

48. Hodgson H, De La Peña R, Stephenson MJ, Thimmappa R, Vincent JL, Sattely ES, et al.. Identification of key enzymes responsible for protolimonoid biosynthesis in plants: Opening the door to azadirachtin production. *Proc Natl Acad Sci U S A*. 116:17096–1042019;

49. pf1600_structural_tmap. https://mandelbrot-project.github.io/pf_1600_datanote/data/outputs/structural/tmap/pf1600_structural_tmap.html Accessed 2022 Nov 11.

50. UCSD Computational Mass Spectrometry Website. https://gnps.ucsd.edu/ProteoSAFe/status.jsp?task=ee2e8e9cf8214f48ab6ee01df652a3f2 Accessed 2022 Nov 11.

51. ReDU Documentation. https://mwang87.github.io/ReDU-MS2-Documentation/ Accessed 2022 Nov 11.

52. Jarmusch AK, Aron AT, Petras D, Phelan VV, Bittremieux W, Acharya DD, et al.. A Universal Language for Finding Mass Spectrometry Data Patterns. *bioRxiv*. :2022.08.06.5030002022;

53. UCSD Computational Mass Spectrometry Website. https://proteomics2.ucsd.edu/ProteoSAFe/status.jsp?task=3197f70bed224f9ba6f59f62906839e9 Accessed 2022 Nov 11.

54. Allard P; Gaudry A; Quirós-Guerrero L; Rutz A; Dounoue-Kubo M; N Walker TW; Defossez E; Long C; Grondin A; David B; Wolfender J. Supporting data for "Open and re-usable annotated mass spectrometry dataset of a chemodiverse collection of 1,600 plant extracts." GigaScience Database 2022. http://dx.doi.org/10.5524/102323

Figure 1

Click here to access/download;Figure;figure_1.pdf

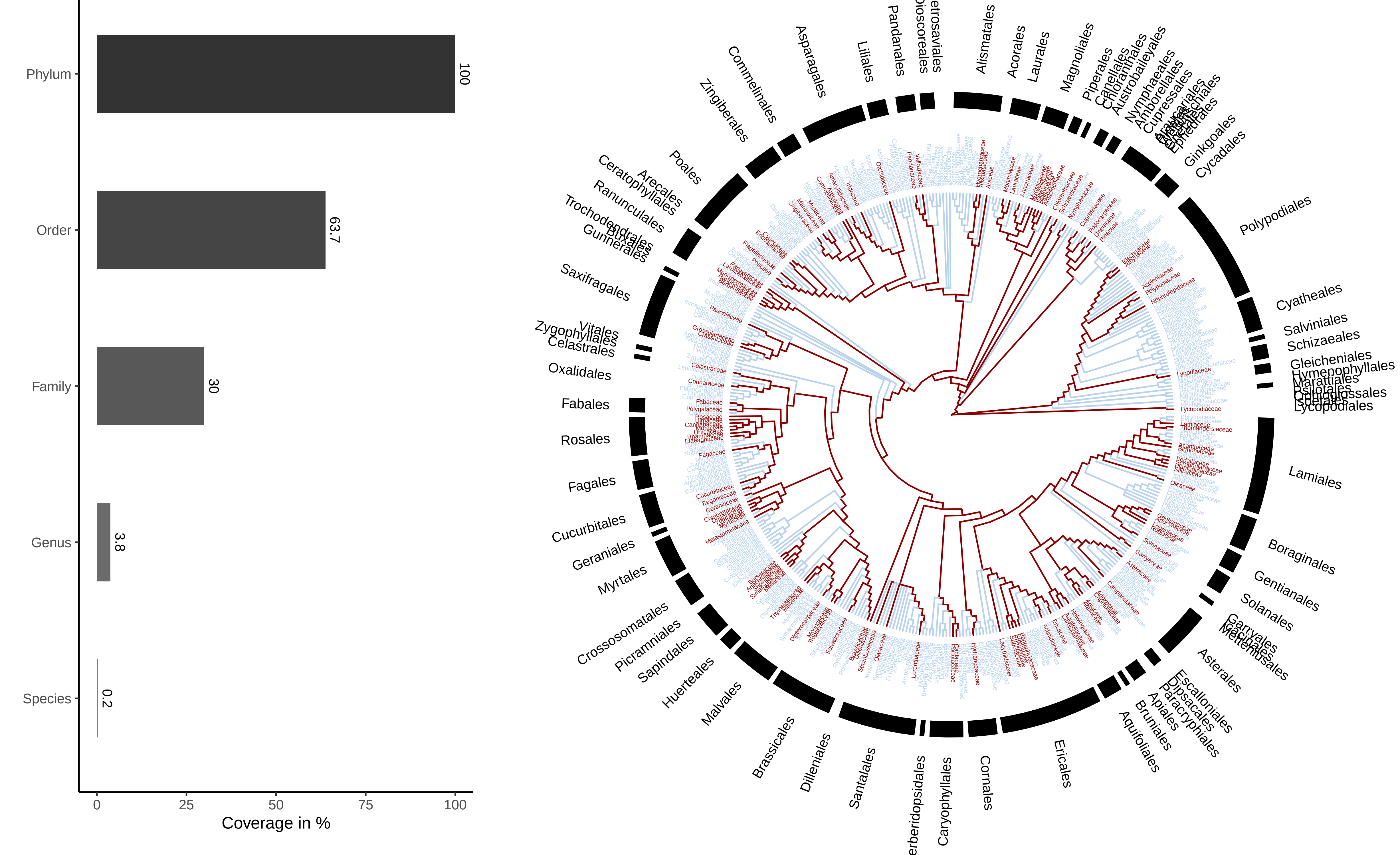

Figure 2

Click here to access/download;Figure;figure_2.pdf

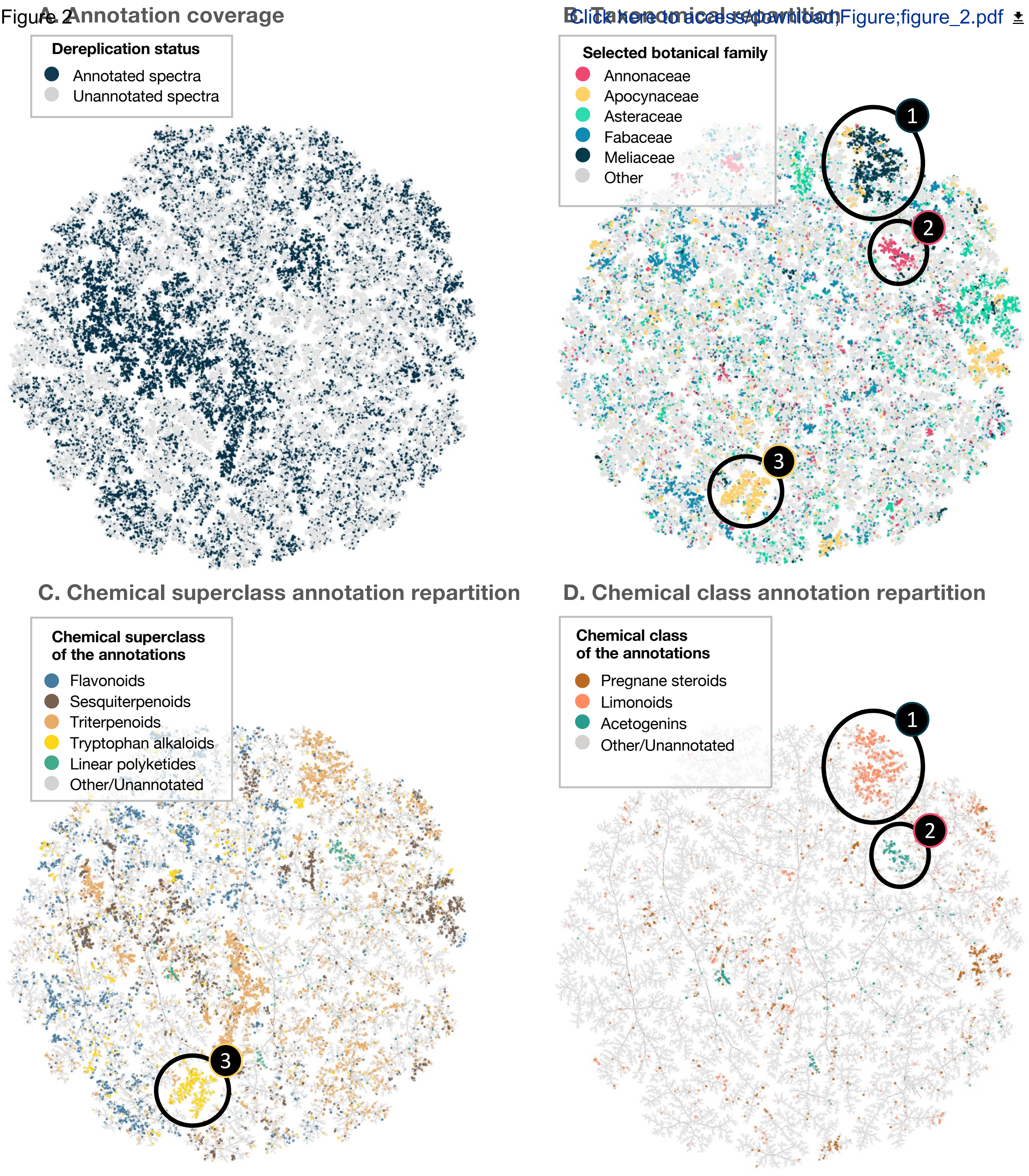

Figure 3

Click here to access/download;Figure;figure_3.pdf

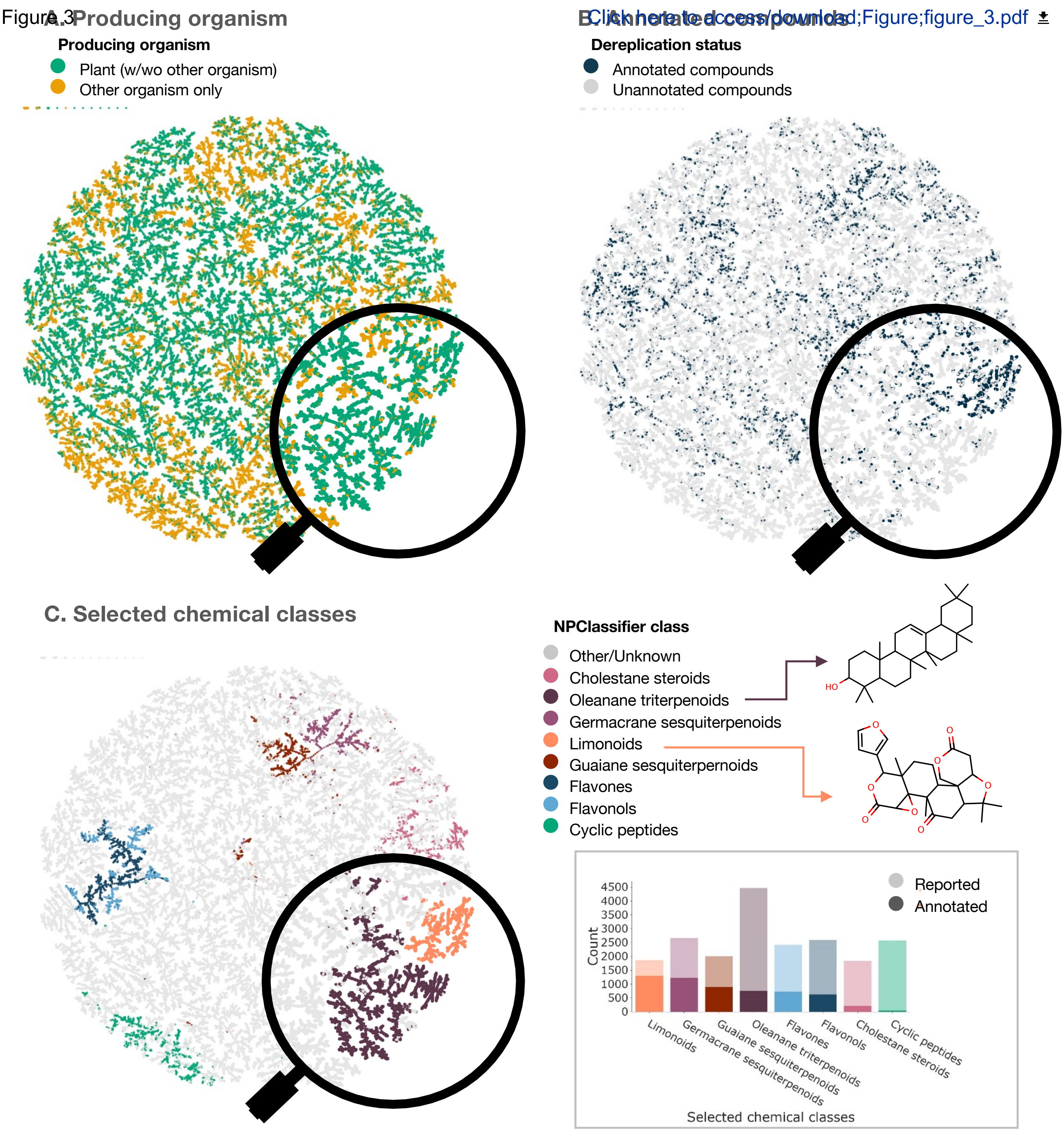

Supplement: giac124_GIGA-D-22-00126_Revision_2 [file giac124_giga-d-22-00126_revision_2.pdf]
