# Supplementary material for: Open and reusable annotated mass spectrometry dataset of a chemodiverse collection of 1,600 plant extracts
Source: Gigascience. 2023 Jan 18;12:giac124. doi: 10.1093/gigascience/giac124 (PMC9845059; doi:10.1093/gigascience/giac124)
Supplement: giac124_Response_to_Reviewer_Comments_Original_Submission [file giac124_response_to_reviewer_comments_original_submission.pdf]

Author's Response To Reviewer Comments

Close

Reviewer reports:

Reviewer #1: This Data Note describes a LC-MS/MS dataset acquired from 1,600 plant extracts, which are a part of the Pierre Fabre extract collection. The entire data is available through MassIVE, a proper public repository for LC-MS/MS-based metabolomics dataset, with an organized metadata. The background history and technical method are well described. All the curated data are accessible through hyperlinks. Large-scale mass spectrometry data on plant specialized metabolites cannot be found commonly, so this Data Note have its own value, and it will be beneficial to the other researchers in relevant fields. Thus, I recommend the publication of this Data Note.
I only have minor comments as below:

- Abstract should clarify that this dataset came from LC-MS/MS.

Done in “After describing the taxonomic coverage of this collection, we present the results of its liquid chromatography high-resolution mass spectrometric profiling and the exploitation of these profiles using computational solutions.” and keyword LCMS

- If the authors can give some examples how (I mean technically) this dataset could be re-used, it will be beneficial to the readers. Recently some softwares enhancing data re-use in metabolomics have launched, so maybe they are worth to be mentioned.

We thank the reviewer for this suggestion. To exemplify and showcase reuse possibilities we moved the Data Availability section to the main text, renamed it Data availability and reusability and presented a series of advanced reused possibilities using notably, ReDu and MassQL. See main text.

- The authors used the word 'exploitation' several times, but it is not clear what it means exactly. Please replace it with a word describing what was exactly provided from the data curation.

We changed two occurrences of exploitation for exploration. We employed exploitation in the sense that data can be seen as a resource and thus be exploited in several manners.

- Introduction: As a researcher outside Europe, I cannot understand what the fact that the PFL collection was 'registered' at the European Commission means; registered for what, or on which list? Please specify this.

We thank the reviewer for this comment and clarified the implication accordingly in the main text. See “The PFL collection, which is among the largest collection plant samples worldwide with over 17,000 unique samples, was registered on April 1, 2020 at the European Commission under the accession number 03-FR-2020. This official registration recognizes the legality of the access and management process. In detail it means that the collection meets the criteria set out in the EU ABS Regulation which implements at the European level the requirements of the Nagoya Protocol regarding access to genetic resources and the fair and equitable sharing of benefits arising from their utilization. [14] To date three european collections are recognized (https://ec.europa.eu/environment/nature/biodiversity/international/abs/pdf/Register%20of%20Collections.pdf).”

- In discussion on the taxonomic coverage, the absolute size of each taxon (order and family) should be considered. E.g. In the World Flora Online, 5615 species are enlisted in Sapindales while 481 species in Cyatheales.

Following this comment and to further document the coverage we prepared two interactive plots available for the readers to explore 1) the coverage according to the numbers of families per order 2) the coverage according to

the numbers of genus per family. See also the main text : “Interactive bar plots are available for the inspection of the coverage of orders (by families) and of families (by genus).”

- The method for MS/MS molecular networking is described but the resulting network is not curated in the main text. Thus, I doubt if the method needs to be included here.

We are indeed not depicting the molecular network in this paper for several reasons. First it is very massive (>110000 nodes) and thus static depictions are hardly usable. The second reason is linked to the previous one, indeed it is because of the size of such a network that we employed TMAP based visualization of the spectral space and these figures are used in the paper. We however believe that the interactive exploration of molecular networks is very efficient to navigate through chemical families. We thus added a “ready to explore” cytoscape file corresponding to a fully annotated molecular network and the following line in the main text : “A Cytoscape file corresponding to the full molecular network mapped with a color layout corresponding to NPClassifier chemical classification, the experimental and theoretical spectral matches as well as a feature table grouped at the family level is available through the following MassIVE repository link.”

- It is interesting that flavonoids showed relatively low coverage because it is one of the most commonly distributed classes of phytochemicals. Could the authors discuss on this?

Here we invite the reviewer to have a closer look at the following interactive barplot https://mandelbrot-project.github.io/pf_1600_datanote/barchart_superclass.html (also linked in the main text). It can be observed that flavonoids are indeed among the large superclass in terms of diversity. The bar plot indicates that the coverage at this specific class is slightly over 41%, larger superclass such as triterpenoids (also very widespread) indicate a coverage of 38 % so overall quite comparable and not particularly low for this specific class of compounds.

- In page 13, some subfigures are indexed wrong. 1B and 1C should be revised to 3B and 3C, respectively.

Thank you for pointing out this error. It has been corrected in the main text.

- Some citations, especially patents, need more bibliographic information.

Thanks for spotting these errors. The bibliography should now be up to date.

Reviewer #2: Allard et al. present a public dataset of plant extracts and their initial exploration using computational tools to mine this dataset. The authors aim to frame this as just the beginning in extracting all the discovery potential from this dataset.

Main Comments

Please include a citation for the MEMO tool.

The citation for the MEMO tool is appearing on page 9. See “Applied to the current plant extract collection, MEMO allows efficient reduction of the observable batch-effect and clustering samples according to their content [18].”

I would recommend a more detailed description of the usage of the TMAP visualization. For example, what exactly is the score of peaks and losses between MS/MS spectra. Was it the aligned cosine used in the molecular networking or something more akin to a shared peak count?

We thank the reviewer for this question and have detailed the process of the TMAP construction both for the spectral and structural based TMAPs. See main text e.g. “In addition to the MN, spectra were organized using TMAP visualization [20]. Briefly, a minimum spanning tree is built from a network of spectral similarity. For this, spectra were first translated to documents (two decimals were used, i.e. a peak at 100.3897 would be translated as “peak@100.39”) using matchms and spec2vec packages (Huber et al. 2020; Huber et al. 2021), with calculation of neutral losses (of up to 400 m/z) to the precursor. The spectral documents were then hashed using

the MinHash scheme and indexed in an LSH forest that was used to generate the TMAP visualization based on the presence/absence of peaks and losses in spectra [21] and [24].”

Additionally, it seems implied that the TMAP is based upon the clustered MS/MS spectra produced by the MZMine tool, but should be made more explicit.

Thank you. This was specified in the main text and a link was added. See “Here the spectral list corresponding to the aligned feature table produced by MzMine was employed.” Please note that the links to the input file are equally present in the TMAP generating scripts.

The text for the section "Visualization of the metabolite annotations" is rather confusing and I actually cannot parse out the specific meaning here. I think the message should be related to Figure 3, where a TMAP is created utilizing the structure similarity of the putative annotations. However, the mixing of references to Figure 1 is rather confusing and I would recommend sticking with Figure 3's visualizations in carrying the story forward.

Indeed, this was also mentioned by the other reviewer. We corrected the reference to the Figure and the text should now read better.

Minor Comments

Correction in abstract, should be: "Researchers interested in the exploitation of large
and chemodiverse extracts collections should use elaborate strategies to
efficiently tackle the chemical complexity and access these structures. "

Here we wanted to use “elaborate” as a verb. We changed the sentence to “Researchers interested in the exploration of large and chemodiverse extracts collections should thus establish strategies aiming to efficiently tackle such chemical complexity and access these structures.”

Overall, I believe the authors limit their claims on this manuscript which is much in line with the results presented. It is not meant to be a final analysis but just the start. I do think some of the results language and presentation needs to be tightened up, as some parts as noted above are hard to comprehend and as a reader am a little confused as the message. As the authors are already limiting the scope to presenting the data, metadata (which is honestly very complete), and preliminary analysis, I think it would be good to sharpen the conclusions of the initial analysis since the merits of this paper does not rest upon an extensive results section.

We thank the reviewer for this comment. We have added a Data availability and reusability section in the main text and before the conclusion. This section showcases some reuse scenarios for this dataset and should hopefully clarify the message of the paper : “Here is a large metabolomics dataset which was fully annotated by the authors. Scripts, methods and results are made available to the community. Now it is open to further reanalysis. We are very much looking forward for such reutilisation by others !”

Close
