# Supplementary material for: Open and reusable annotated mass spectrometry dataset of a chemodiverse collection of 1,600 plant extracts
Source: Gigascience. 2023 Jan 18;12:giac124. doi: 10.1093/gigascience/giac124 (PMC9845059; doi:10.1093/gigascience/giac124)
Supplement: giac124_Reviewer_1_Report_Original_Submission — Kyo Bin Kang -- 6/17/2022 Reviewed [file giac124_reviewer_1_report_original_submission.pdf]

Reviewer Report

Title: Open and re-usable annotated mass spectrometry dataset of a chemodiverse collection of 1,600 plant extracts.

Version: Original Submission **Date:** 6/17/2022

Reviewer name: Kyo Bin Kang

Reviewer Comments to Author:

This Data Note describes a LC-MS/MS dataset acquired from 1,600 plant extracts, which are a part of the Pierre Fabre extract collection. The entire data is available through MassIVE, a proper public repository for LC-MS/MS-based metabolomics dataset, with an organized metadata. The background history and technical method are well described. All the curated data are accessible through hyperlinks. Large-scale mass spectrometry data on plant specialized metabolites cannot be found commonly, so this Data Note have its own value, and it will be beneficial to the other researchers in relevant fields. Thus, I recommend the publication of this Data Note.I only have minor comments as below:- Abstract should clarify that this dataset came from LC-MS/MS.- If the authors can give some examples how (I mean technically) this dataset could be re-used, it will be beneficial to the readers. Recently some softwares enhancing data re-use in metabolomics have launched, so maybe they are worth to be mentioned.- The authors used the word 'exploitation' several times, but it is not clear what it means exactly. Please replace it with a word describing what was exactly provided from the data curation.- Introduction: As a researcher outside Europe, I cannot understand what the fact that the PFL collection was 'registered' at the European Commission means; registered for what, or on which list? Please specify this.- In discussion on the taxonomic coverage, the absolute size of each taxon (order and family) should be considered. E.g. In the World Flora Online, 5615 species are enlisted in Sapindales while 481 species in Cyatheales.- The method for MS/MS molecular networking is described but the resulting network is not curated in the main text. Thus, I doubt if the method needs to be included here.- It is interesting that flavonoids showed relatively low coverage because it is one of the most commonly distributed classes of phytochemicals. Could the authors discuss on this?- In page 13, some subfigures are indexed wrong. 1B and 1C should be revised to 3B and 3C, respectively.- Some citations, especially patents, need more bibliographic information.

Level of Interest

Please indicate how interesting you found the manuscript: Choose an item.

Quality of Written English

Please indicate the quality of language in the manuscript: Choose an item.

Declaration of Competing Interests

Please complete a declaration of competing interests, considering the following questions:

- Have you in the past five years received reimbursements, fees, funding, or salary from an organisation that may in any way gain or lose financially from the publication of this manuscript, either now or in the future?
- Do you hold any stocks or shares in an organisation that may in any way gain or lose financially from the publication of this manuscript, either now or in the future?
- Do you hold or are you currently applying for any patents relating to the content of the manuscript?
- Have you received reimbursements, fees, funding, or salary from an organization that holds or has applied for patents relating to the content of the manuscript?
- Do you have any other financial competing interests?
- Do you have any non-financial competing interests in relation to this paper?

If you can answer no to all of the above, write 'I declare that I have no competing interests' below. If your reply is yes to any, please give details below.

I declare that I have no competing interests.

I agree to the open peer review policy of the journal. I understand that my name will be included on my report to the authors and, if the manuscript is accepted for publication, my named report including any attachments I upload will be posted on the website along with the authors' responses. I agree for my report to be made available under an Open Access Creative Commons CC-BY license (http://creativecommons.org/licenses/by/4.0/). I understand that any comments which I do not wish to be included in my named report can be included as confidential comments to the editors, which will not be published.

Choose an item.

To further support our reviewers, we have joined with Publons, where you can gain additional credit to further highlight your hard work (see: https://publons.com/journal/530/gigascience). On publication of this paper, your review will be automatically added to Publons, you can then choose whether or not to claim your Publons credit. I understand this statement.

Yes Choose an item.
