# Supplementary material for: Open and reusable annotated mass spectrometry dataset of a chemodiverse collection of 1,600 plant extracts
Source: Gigascience. 2023 Jan 18;12:giac124. doi: 10.1093/gigascience/giac124 (PMC9845059; doi:10.1093/gigascience/giac124)
Supplement: giac124_Reviewer_2_Report_Original_Submission — Mingxun Wang -- 6/23/2022 Reviewed [file giac124_reviewer_2_report_original_submission.pdf]

Reviewer Report

Title: Open and re-usable annotated mass spectrometry dataset of a chemodiverse collection of 1,600 plant extracts.

Version: Original Submission **Date:** 6/23/2022

Reviewer name: Mingxun Wang

Reviewer Comments to Author:

Allard et al. present a public dataset of plant extracts and their initial exploration using computational tools to mine this dataset. The authors aim to frame this as just the beginning in extracting all the discovery potential from this dataset.Main CommentsPlease include a citation for the MEMO tool.I would recommend a more detailed description of the usage of the TMAP visualization. For example, what exactly is the score of peaks and losses between MS/MS spectra. Was it the aligned cosine used in the molecular networking or something more akin to a shared peak count? Additionally, it seems implied that the TMAP is based upon the clustered MS/MS spectra produced by the MZMine tool, but should be made more explicit.The text for the section "Visualization of the metabolite annotations" is rather confusing and I actually cannot parse out the specific meaning here. I think the message should be related to Figure 3, where a TMAP is created utilizing the structure similarity of the putative annotations. However, the mixing of references to Figure 1 is rather confusing and I would recommend sticking with Figure 3's visualizations in carrying the story forward.Minor CommentsCorrection in abstract, should be: "Researchers interested in the exploitation of largeand chemodiverse extracts collections should use elaborate strategies toefficiently tackle the chemical complexity and access these structures. "Overall, I believe the authors limit their claims on this manuscript which is much in line with the results presented. It is not meant to be a final analysis but just the start. I do think some of the results language and presentation needs to be tightened up, as some parts as noted above are hard to comprehend and as a reader am a little confused as the message. As the authors are already limiting the scope to presenting the data, metadata (which is honestly very complete), and preliminary analysis, I think it would be good to sharpen the conclusions of the initial analysis since the merits of this paper does not rest upon an extensive results section.

Level of Interest

Please indicate how interesting you found the manuscript: Choose an item.

Quality of Written English

Please indicate the quality of language in the manuscript: Choose an item.

Declaration of Competing Interests

Please complete a declaration of competing interests, considering the following questions:

- Have you in the past five years received reimbursements, fees, funding, or salary from an organisation that may in any way gain or lose financially from the publication of this manuscript, either now or in the future?
- Do you hold any stocks or shares in an organisation that may in any way gain or lose financially from the publication of this manuscript, either now or in the future?
- Do you hold or are you currently applying for any patents relating to the content of the manuscript?
- Have you received reimbursements, fees, funding, or salary from an organization that holds or has applied for patents relating to the content of the manuscript?
- Do you have any other financial competing interests?
- Do you have any non-financial competing interests in relation to this paper?

If you can answer no to all of the above, write 'I declare that I have no competing interests' below. If your reply is yes to any, please give details below.

None

I agree to the open peer review policy of the journal. I understand that my name will be included on my report to the authors and, if the manuscript is accepted for publication, my named report including any attachments I upload will be posted on the website along with the authors' responses. I agree for my report to be made available under an Open Access Creative Commons CC-BY license (http://creativecommons.org/licenses/by/4.0/). I understand that any comments which I do not wish to be included in my named report can be included as confidential comments to the editors, which will not be published.

Choose an item.

To further support our reviewers, we have joined with Publons, where you can gain additional credit to further highlight your hard work (see: https://publons.com/journal/530/gigascience). On publication of this paper, your review will be automatically added to Publons, you can then choose whether or not to claim your Publons credit. I understand this statement.

Yes Choose an item.
