# Supplementary material for: Open and reusable annotated mass spectrometry dataset of a chemodiverse collection of 1,600 plant extracts
Source: Gigascience. 2023 Jan 18;12:giac124. doi: 10.1093/gigascience/giac124 (PMC9845059; doi:10.1093/gigascience/giac124)
Supplement: giac124_Authors_Response_To_Reviewer_Comments [file giac124_authors_response_to_reviewer_comments.pdf]

Author's Response To Reviewer Comments

Close

Dear GigaScience editorial office,

We have integrated and responded to the formatiing comments on this last revised version.
Looking forward to read from you,

Best regards,

Pierre-Marie Allard

Close
